# Frequency tagged multifocal pupillary response fields identify age-related macular degeneration and diabetic retinopathy

**DOI:** 10.3389/fnins.2025.1653938

**Published:** 2026-02-10

**Authors:** Laure Trinquet, Thierry David, Frédéric Chavane, Jean Lorenceau, Fréderic Matonti

**Affiliations:** 1 Faculté des Sciences Médicales et Paramédicales, Aix-Marseille-University, Marseille, France; 2 Institut des Neurosciences de la Timone, Aix-Marseille University, CNRS UMR7289, Marseille, France; 3 Department of Ophthalmology, Hospital Timone, Marseille, France; 4 Université Paris Cité, INCC UMR 8002, CNRS, Paris, France; 5 Centre Monticelli Paradis, Marseille, France; 6 Clinique Juge, Almaviva Santé, Marseille, France

**Keywords:** diabetic retinopathy, frequency tagging, macular degeneration, pupillary fields, retina

## Abstract

**Objectives:**

To quickly characterize multifocal Pupillary Response Fields (mPRF) using frequency tagging and to identify pupillary biomarkers of age-related macular degeneration (AMD) and diabetic retinopathy (DR).

**Methods:**

Participants with AMD (*N* = 74), DR (*N* = 56), and healthy controls (HP, N = 62) underwent standard ophthalmologic assessments together with a multifocal Pupillary Frequency Tagging test (mPFT). The mPFT test comprised 9 retinal regions whose luminance were sinusoidally modulated at incommensurate temporal frequencies so as to elicit sustained pupillary oscillations over 45 s of fixation.

**Analyses:**

The recorded pupillary traces were corrected for blinks and transient artifacts. Features of pupillary dynamics and eye-movements, − eye instability during fixation, pupil light reflex, and spectral components of pupil oscillations - were compared across groups. Statistical analyses were performed for each feature separately using Student t-tests and Cohen’s *d*. The Area under the Curve (AUC) of the Receiver Operating Characteristics (ROC) was computed for different combinations of the extracted features. Pearson’s correlation was used to compare spectral power with other functional measures.

**Results:**

Multifocal Pupillary Response Fields (mPRF) derived from regional spectral power and phase distributions differed significantly between patients and controls, in accordance with the characteristics of each pathology: decreased power for central and paracentral sectors in AMD, diffuse defects in DR. AUCs of ROC performed with relevant features achieved excellent sensitivity (>0.9) and specificity (>0.9) in classifying patients from healthy subjects.

**Conclusion:**

Fast, objective, and easily recorded, mPRF assessments evaluate the functional integrity of retino-pupillary circuits, providing spatiotemporal bio-signatures selective for maculopathies and retinopathies.

## Introduction

Ophthalmic diseases such as age-related macular degeneration (AMD) and diabetic retinopathy (DR) are among the leading causes of visual impairment and blindness worldwide, posing major public health and socio-economic challenges (Lim et al., 2012; Teo et al., 2021).

AMD primarily affects central vision through degeneration of macular photoreceptors (Curcio, 1996; Jones et al., 2016), evolving from early drusen deposits to atrophic or neovascular late-stage forms. In contrast, DR is a diabetic microangiopathy involving ischemia, neovascularization, and macular edema, which ultimately impairs photoreceptor function (Barber, 2003; Lynch and Abràmoff, 2017). Despite therapeutic advances, these diseases remain difficult to manage effectively and require early, objective, and functionally informative assessment tools.

Current functional visual assessments rely on subjective tests such as visual acuity (VA) and standard automated perimetry (SAP), which are limited by their dependence on patient cooperation, sustained attention, and cognitive status. These factors result in high test–retest variability, particularly in elderly or cognitively vulnerable individuals (Spry et al., 2001; Mendieta et al., 2021; Heijl et al., 2022). Structural imaging tools such as optical coherence tomography (OCT) provide high-resolution views of retinal morphology and thickness. However, they offer limited insight into functional visual performance, especially in early or discordant cases, underscoring the need for objective and spatially resolved functional assessments (Maloca et al., 2019).

Pupillometry emerges as a promising solution, enabled by improved understanding of the neural circuits underlying pupil control and progress in eye-tracking technology. Previously, numerous studies established that cones and rods together shape the characteristics of pupillary responses under a variety of chromatic and luminance conditions and that pupil responses closely match visual sensitivity and perception (Alpern and Campbell, 1962; Alpern et al., 1963; Campbell and Gubisch, 1966; Young and Kennish, 1993). Pupil responses to light are thus a proxy well suited to assess visual defects in ophthalmic diseases. This view is reinforced by the discovery of intrinsically photosensitive retinal ganglion cells (ipRGCs) expressing melanopsin together with the identification of their retinal connectivity and circuits (Provencio et al., 1998; Berson et al., 2002; Dacey et al., 2005; Barrionuevo et al., 2014; Zele et al., 2019; Zele and Gamlin, 2020; Schmid et al., 2000; Spitschan and Woelders, 2018; Spitschan, 2019). The axons of ipRGCs project to the pretectal olivary nucleus, which in turn relays signals to the Edinger–Westphal nucleus and the ciliary ganglion, ultimately triggering iris constriction (Szabadi, 2018; Barrionuevo et al., 2023; Szabadi and Bradshaw, 1996). ipRGCs integrate direct blue-light input (peak ~480 nm) and indirect signals from rods and cones via bipolar and amacrine cells (Hattar, 2002; Chen et al., 2011; Lucas et al., 2014; Zhao et al., 2014; Wong, 2018). This dual input drives a fast pupil light reflex (PLR, 300–1,000 ms) relying on rods and cones responses and a slow sustained post-illumination pupil response (PIPR), reflecting intrinsic ipRGC activity (Kankipati et al., 2010; Adhikari et al., 2015; Zele et al., 2019). The fast pupil response to light involves photoreceptors and bipolar cells, can follow sinusoidal luminance modulations over a wide range - ~ 0.1 to 10 Hz although with decreased amplitude beyond ~4 Hz- (Clarke et al., 2003; Barrionuevo et al., 2014), and can be evoked by a wide range of wavelengths targeting all photoreceptors. Research using silent substitution methods used to isolate the respective contributions of cones and rods (Donner and Rushton, 1959; Spitschan and Woelders, 2018), remain technically demanding and less suited to clinical applications.

Despite its potential, conventional pupillometry still faces methodological and practical barriers to clinical application. Most studies rely on eliciting PLRs using brief pulses of light, either full-field or as localized flashes presented sequentially across the visual field (Maddess et al., 2009; Sabeti et al., 2014, 2015; Kelbsch et al., 2020). These paradigms typically require 5–10 min of dark adaptation, followed by repeated stimulations interleaved with recovery periods (Kelbsch et al., 2019). These procedures are time-consuming, fatiguing, and often poorly tolerated, especially in patients with photosensitivity or reduced attentional capacity.

PLR analysis typically focuses on latency, amplitude, and constriction speed (Bremner, 2012; Jendritza et al., 2024). While abnormalities have been documented in various ocular diseases (Wilhelm and Wilhelm, 2003; Bremner, 2004; Bremner and Smith, 2006; Carle et al., 2014; Sabeti et al., 2014; Park et al., 2017; Najjar et al., 2018; Chougule et al., 2019; Rukmini et al., 2019; Pinheiro and Da Costa, 2021; Rai et al., 2021; Decleva et al., 2023), global measures lack spatial resolution and cannot localize region-specific dysfunction, limiting their value in heterogeneous diseases such as AMD and DR. While chromatic paradigms based on PIPR or silent substitution can isolate photoreceptor-specific responses (Spitschan and Woelders, 2018), their complexity limits clinical implementation (Kelbsch et al., 2019).

To overcome these limitations, we developed a novel pupillometric approach: Multifocal Pupillary Frequency Tagging (mPFT). This method provides fast (1 min per eye), objective, and spatially resolved assessment of retinal function (Ajasse et al., 2022; Stelandre et al., 2023; Lorenceau et al., 2024). Unlike classical PLR-based protocols, mPFT simultaneously modulates the luminance of nine predefined retinal regions, each with a distinct sinusoidal temporal frequency, incommensurable with the others. In healthy participants, the luminance modulation of each sector at its associated frequency should entrain a modulation of pupil size exclusively at this particular frequency. The resulting multiplexed pupil response reflecting the respective contributions of the 9 Frequency-Of-Interest (FOIs) is then analyzed with a Fourier transform to extract the sector-specific spectral components. The spectral power at FOIs is then used to generate multifocal Pupillary Response Fields (mPRF) showing the relative contribution of each sector (Lorenceau et al., 2024).

mPFT requires no verbal response, and is fully compatible with clinical settings. Unlike conventional paradigms that rely on sequential presentations, mPFT captures all regions simultaneously. Previous studies have shown that mPRFs obtained with this technique correlate with localized functional deficits in conditions such as glaucoma, optic neuropathies, and retinal dystrophies (Ajasse et al., 2022; Stelandre et al., 2023; Lorenceau et al., 2024).

In this study, we applied mPFT to two major retinal diseases: late AMD and DR. We hypothesized that AMD would produce selective deficits in central/paracentral regions, reflecting macular photoreceptor loss, while DR would induce diffuse alterations due to widespread vascular remodeling and macular edema. We also investigated whether eye movement behavior during stimulation, such as blink rate, fixation stability, and intrusive saccades, could serve as additional functional markers. Finally, we assessed the diagnostic performance of mPFT-derived features using Receiver Operating Characteristic (ROC) curves and corresponding Area Under the Curve (AUC) values. We also explored potential correlations between mPFT metrics, visual acuity (VA), and other clinical factors, such as the presence of cataract.

## Method

### Participants

This monocentric, cross-sectional study was conducted at the Monticelli Paradis Center (Marseille, France), between October 2022 and December 2023, in accordance with the Declaration of Helsinki and approved by a French Ethics Committee (“Comité de Protection des Personnes Ile-de-France III,” ID-RCB 2022-A00708-35). Participants were informed of the objectives of the study and gave their written informed consent to perform the pupillary tests.

The studied eyes, selected according to the inclusion criteria defined in the protocol, were grouped as follows (Table 1).

**Table 1:** Demographics of participants, studied eyes by pathology, visual acuity.

Healthy participants	*N*	Male	Female	Mean age	Dark eyes	Light eyes
	68	25	43	53.35 ± sd 14.5	71	61
LogMAR	>1.1	0.8–1.1	0.5–0.7	0.3–0.4	0.2	<0.2
Number of eyes	0	0	0	0	0	132
Cataract	LOCS III = 0	LOCS III = 1	LOCS III = 2	ICP		
Number of eyes	121	8	0	3		

Age-related macular degeneration	*N*	Male	Female	Mean age	Dark eyes	Light eyes
	97	38	59	76.14 ± sd 6.3	83	73
LogMAR	>1.1	0.8–1.1	0.5–0.7	0.3–0.4	0.2	<0.2
Number of eyes	7	25	22	30	21	51
Cataract	LOCS III = 0	LOCS III = 1	LOCS III = 2	ICP		
Number of eyes	9	20	28	99		
AMD	Atrophic	Exsudative				
Number of eyes	32	124				

Diabetic retinopathy	*N*	Male	Female	Mean age	Dark eyes	Light eyes
	57	32	25	60.5 ± sd16	83	73
LogMAR	>1.1	0.8–1.1	0.5–0.7	0.3–0.4	0.2	<0.2
Number of eyes	3	9	13	24	22	42
Cataract	LOCS III = 0	LOCS III = 1	LOCS III = 2	ICP		
Number of eyes	26	16	14	57		
DRSS	DRSS 35	DRSS 43	DRSS 47–53	DRSS 61–55	DRSS 71–75	
Number of eyes	4	19	24	32	34	

Healthy participants (HP), Age-related macular degeneration (AMD), Diabetic retinopathy (DR). SD, indicates standard deviation.

In the group with age-related macular degeneration (AMD), there were 34 eyes with Age-Related Maculopathy (ARM), 32 eyes with atrophic AMD, and 124 eyes with exudative AMD. In the group with Diabetic Retinopathy (DR), 47 eyes presented non-proliferative DR, 66 eyes had proliferative DR, and 71 eyes had a diabetic macular oedema. Healthy Participants (HP) met the following inclusion criteria: Visual acuity above or equal to 8/10 (corresponding to approximately 20/25 Snellen, ~0.1 LogMAR, ~80 ETDRS letters), absence of ophthalmologic pathology and no previous ophthalmologic issues. For the patients groups, exclusion criteria were the presence of another retinal pathology, cataract surgery within the last 6 months, previous eye surgery other than cataract surgery, panphotocoagulation within the last 12 months, intravitreal injection (IVT) within less than 1 month, a major cataract greater than 2 + on the lens opacity classification, previous ocular trauma, a previous diagnosis of optic neuropathy, uveitis, glaucoma, a high vertical slice-to-disk ratio of >0.7, high intraocular pressure, conditions affecting the pupils, including the use of drugs that affect pupillary size or response.

A few participants had to be excluded, either because they presented comorbidities incompatible with the inclusion criteria or, for HP, because of insufficient visual acuity. Twelve data sets (on 435, 2.8%) from the pupil tests were excluded because of a technical issue or because of the poor quality of pupil signals.

### Clinical assessments

All patients underwent a standard ophthalmologic examination, including measurement of visual acuity (VA), Optical Coherence Tomography (OCT), intraocular pressure (IOP) measurement, fundus examination (FE) and recordings of current and past treatments. Healthy participants underwent functional assessments of visual acuity, intraocular pressure, and fundus examination. However, for practical reasons, an OCT examination was performed only for 50 participants. Participants completed a questionnaire (Supplementary Text 1) before and after the pupillary test to evaluate their experience, including fatigue, discomfort and perceived difficulty.

### Stimuli and apparatus

The details of the mPFT stimulus and the analysis of pupillary signals have been described in detail in a previous article, (Lorenceau et al., 2024) and are only briefly summarized here.

**Figure 1:**
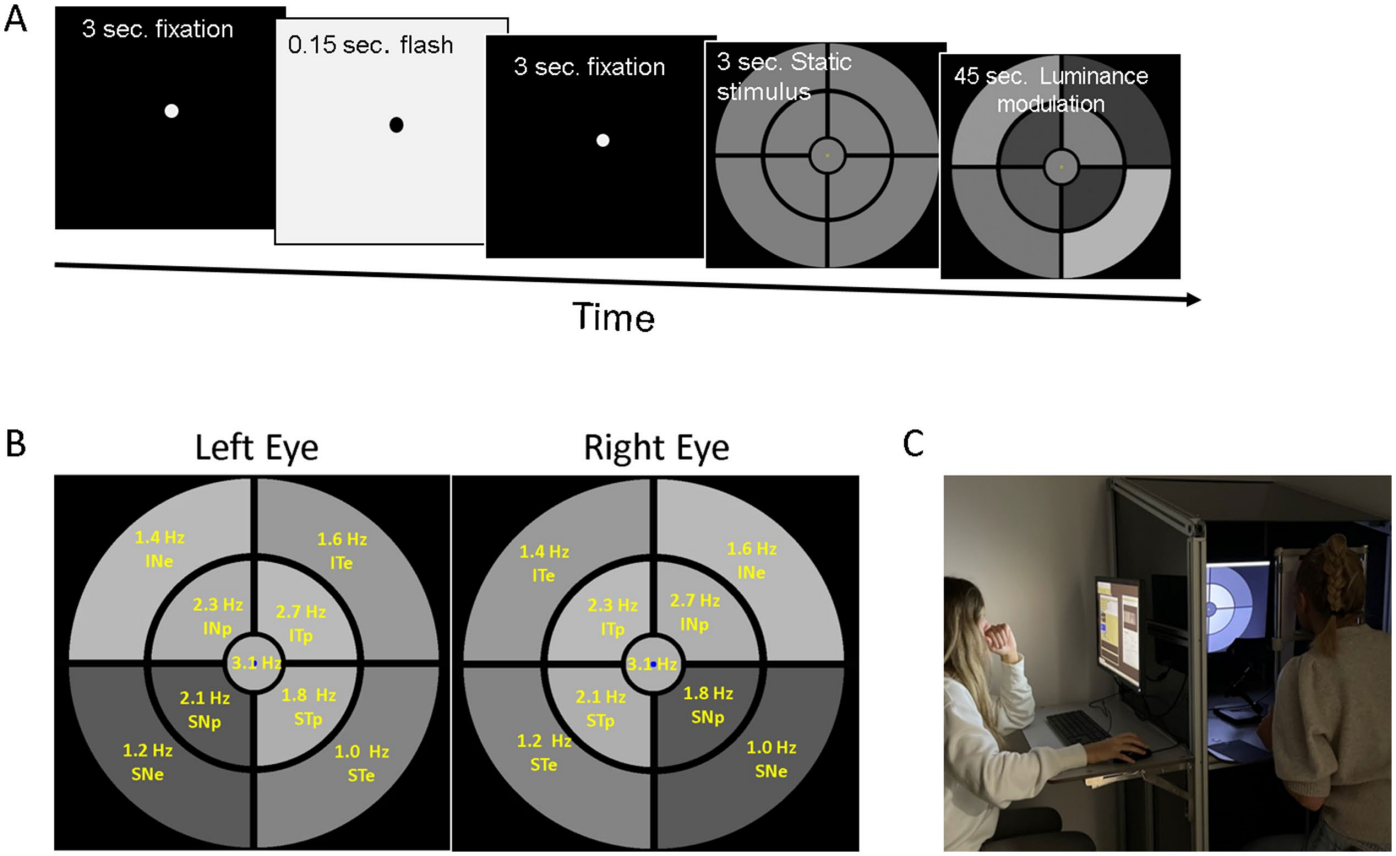
**(A)** Time course of a trial: 3 s fixation followed by a flash eliciting a pupil light reflex, followed by 3 s of fixation, and 3 s of the static display to allow the pupil to adapt to the mean luminance, followed by 45 s of luminance oscillations for the 9 sectors. **(B)** Stimuli used in the study. Each stimulus was presented to the right or left eye. Yellow texts indicate the temporal modulation frequency used for each sector together with their retinal projection: SNe supero-nasal eccentric, STe supero-temporal eccentric; ITe infero-temporal eccentric, INe infero-nasal eccentric, SNp supero-nasal paracentral, STp supero-temporal paracentral; ITp infero-temporal paracentral, INp infero-nasal paracentral, and central. **(C)** Photograph of the “Eye Box” used to isolate the participants (right), where the remote eye-tracker and the stimulus are visible.

The mPFT stimulus covers about 40° of visual angle and comprises 9 sectors of different sizes arranged to stimulate foveal, parafoveal, and eccentric regions (Figure 1). During mPFT (45 s.), each sector is periodically modulated in luminance at a specific temporal modulation frequency (TMF), incommensurate with the TMFs of other sectors. The TMFs of mPFT were 1.00, 1.25, 1.39, 1.58, 1.81, 2.14, 2.31, 2.73, and 3.16 Hz. The coupling between a sector and a TMF is held constant and is the same for the right and left eye. These TMFs correspond to the Frequencies of Interest (FOIs) analyzed thereafter. Supplementary Video 1 shows an excerpt of the mPFT stimulus. The luminance modulation amplitude was identical for all sectors, with a maximum of 100 cd/m^2^ and a minimum of 20 cd/m^2^.

The mPFT stimulus was displayed on a conventional monitor (BenQ GW2486TC, 1,024 × 768 × 8 bits refreshed at 60 Hz) placed at 57 cm from the eyes. Monocular eye movements and pupil size were recorded at 500 Hz with a Live Track Lightning remote eye tracker (Cambridge Research System Ltd., Rochester, United Kingdom) placed at 30 cm from the eyes. Recordings were down-sampled to 60 Hz for further analyses. The display and the eye-tracker were controlled by custom software (Jeda) running under Windows 10 (Microsoft Ltd., Redmond, Washington, United States). A chinrest was used to stabilize the participants’ eyes relative to the eye tracker. The display, eye tracker and chinrest were installed in an “Eye-Box” designed to isolate the participants and to limit the field of view (Figure 1C). All tests were performed in a dim ambient light.

### Procedure

After obtaining written informed consent from each participant, a short questionnaire was administered to assess his/her general state (fatigue, existence of treatments, etc.) during which he/she adapted to the dim ambient light of the testing room. The participant was then positioned for the recording session and a 5-point eye calibration was performed once for the whole session, when possible. If precise calibration could not be achieved due to the participant’s condition, a default calibration was used. This did not compromise the validity of the measurements, as pupil size is computed independently of gaze calibration. In addition, relative eye position was sufficient to assess fixation quality and detect intrusive saccades. A session comprised 3 tests of 1 min each, presented to each eye in alternation, resulting in 6 min of recordings. One test was the achromatic mPFT test described herein. A second test used a chromatic version of mPTF not reported here. The last test measured the Pupil Cycle Time (PCT) using a computerized setting (Lamirel et al., 2018; Trinquet et al., 2025). We only report the results for the gray mPFT test herein. For these tests, participants were asked to maintain fixation at the center of the screen (“Look ahead”), marked by a small colored circular fixation disk (0.2°), and to limit blinking as much as possible with no other concurrent task. A brief rest between the different runs was used to change the stimulated eye (right or left in alternation). Overall, a session lasted less than 15 min. Note that running the gray mPFT stimulus only takes 2–3 min.

A run started with a white central fixation target, followed after 1 s by a brief full-screen flash (166 ms) at the maximum screen luminance (165 cd/m2) to elicit a PLR. A dark screen was then displayed for 3 s, followed by the static mPFT homogeneous gray stimulus (51 cd/m2) presented for 3 s to let the pupil adapt to the mean luminance, followed by 45 s of fixation of the temporal luminance modulations of the 9 sectors (Figure 1; Supplementary Video 1).

At the end of a session, participants filled a questionnaire evaluating their feelings during the test (comfort, duration, glare, difficulty).

### Data analyses

All analyses and statistics were performed using custom scripts developed under Matlab R2018b (The MathWorks, Natick, MA, United States).

#### Pupil size

We computed the square of the horizontal pupil diameter delivered by the eye-tracker as an approximation of pupil area, as the horizontal diameter is less susceptible to artifacts than the vertical diameter which can be biased by intermittent occultation by eyelid movements.

#### Data correction

Before computing features characterizing pupillary and eye movements, we detected, removed and replaced blinks and spurious transient data from each recording. Blinks were detected as zeros in pupil traces. A 4-frames (66 ms) window before and after each blink was used. The selected sequence was then replaced by a smoothed linear interpolation between pre and post values.

Fast pupil transients due to imperfect eye-tracking settings, instability of head or eyes during a run, leading to intermittent signal losses, incompatible with the slow pupillary dynamics, were detected and isolated by thresholding pupil velocity. These artefactual segments were replaced using a smoothed linear interpolation. All corrections were quantified for further analysis (Supplementary Table 1; Figure 1). Some recordings could not be analyzed due to technical issues, or excessive blinking. These trials were removed prior to the main analysis. In total, less than 3% of the trials (12/435) were excluded for that reason.

The data correction process can bias the analysis of pupillary oscillations for runs with many blinks or numerous transients. This issue was addressed by identifying and excluding outliers (data points exceeding 3 scaled median absolute deviations from the median) for each variable and each group before statistical analyses.

The corrected pupil traces were then used to quantify relevant pupillary features likely to differ between groups. Table 2 summarizes these features of interest, detailed below.

**Table 2:** Features of interest derived from each monocular run: data corrections, PLR, eye movements, global pupil state, spectral power and phase, relative power between regions.

Data correction	Pupil light reflex (PLR)	Eye-movements during mPFT	Pupil during mPFT	Spectral power during mPFT	Relative power ratios
% of blink corrected data	Base line Pupil Size	sVert & Hor Standard Deviation	Mean pupil size	Power at the 9 FOIs	Left/Right Quadrants
Number of corrected transients	Start Constriction Latency	Ver & Hor Outliers >2 SD of mean positions	Slope of mean pupil size over time	Phase at the 9 FOIs	Up/Down Quadrants
	Pupil Size Peak Constriction		Mean oscillation amplitude		Up/Down and Left/Right
	Peak Constriction Latency		Standard deviation of pupillary oscillations		Peripheral/Paracentral Ratio
	Mean Constriction Speed		Pupil Lag (cross-correlation Stimulus/Pupil)		Central/ All other sectors

Each PLR was quantified by 5 variables: baseline pupil size (Arbitrary Units, A. U.) measured before the PLR inducing flash, latency of starting constriction (*StartLat*), measured as the delay between flash onset and beginning of constriction, minimum pupil size at maximum constriction (*MinPSize*), latency to reach minimum size (*MaxLat*, time elapsed between *StartLat* and *MinPSize*), and mean constriction speed (*Pspeed,* amplitude of pupil constriction divided by the time from *StartLat* and *MaxLat*).

Fixation quality and number of intrusive saccades were estimated by the horizontal and vertical standard deviations of eye positions during a run and the horizontal and vertical number of outliers (eye positions more than 2 Standard Deviation of mean eye position). Global pupil state during mPFT was characterized with 7 variables: slope of a linear fit of pupil size evaluating pupillary drifts over time, mean maximum and minimum pupil size, mean amplitude of oscillatory responses (difference between maximum and minimum pupil size), and standard deviation of pupillary responses. A cross-correlation between stimulus signal and pupil dynamics (*xcor* Matlab function) measured the overall lag of pupil oscillations relative to the stimulus luminance.

The respective contribution of each of the 9 sectors/frequencies to the overall pupil response during mPFT was quantified by a spectral analysis. The signal was first trimmed by 1 s at the beginning and end of the signal, such that 43 s of signal was processed using a Fast Fourier Transform (*fft* Matlab function) to extract the power and phase at each of the 9 Frequencies Of Interest (FOIs). To ensure accurate estimation and to prevent power and phase from spreading across multiple FFT bins, the Frequency Resolution (FR = Sampling Hz/ Number of samples) was adjusted by decreasing the number of samples so that each FOI was an integral multiple of the FR. This was done separately for each FOI.

### Statistics

A Kolmogorov–Smirnov test was first used to assess the normality of the distribution of each variable before applying a Student’s t-test to evaluate the statistical significance of groups’ comparisons. Cohen’s *d* was used to measure effect size. The Area under the Receiving Operating Characteristics Curve (AUC of ROC) was computed using the *fitglm* and *percurve* Matlab functions to determine whether patients and control participants could be classified using different combinations of the features derived from the analyses. Pearson’s correlations were used to compare pupillary variables with other functional measures, or to evaluate age-related effects on variable distributions.

## Results

Before assessing the pupil variables listed in Table 2, we analyzed the demographics of the participants. We found that the mean age of HP was lower than that of the patient groups, and excluded HP participants younger than 35 years (*N* = 7) to attenuate this difference. We also analyzed the distributions of each variable as a function of age. Among the HP group, only 5 variables demonstrated a significant age-related effect, 3 of which were related to the PLR (pupil size at baseline, constriction latency and constriction speed, see Supplementary Table 2). These effects are in line with previous studies, although reports involving a wider age range (e.g., 18–81 years) also noticed significant effects for other PLR characteristics (Kumnick, 1956; Telek, 2018).

### Effects of neuropathies and retinopathies on the components of the pupil response

We detail below the distributions, statistical significance, sensitivity and specificity of the variables characterizing pupil and eye-movement responses for the right eye of the different groups: Healthy Participants (HP), patients with age-related macular degeneration (AMD), diabetic retinopathy (DR). The raw distributions of all variables are available in Supplementary Figures 1–8. The results for the left eyes are overall similar.

#### Pupil light reflex

Table 3 shows the statistics comparing PLR variables (see Method and Table 2) of patients and HP. As it can be seen, all variables are significantly different for DR patients as compared to HP, while the AMD group differ significantly from the HP group for 3 out of the 6 variables (*MinPsize, MaxLat and StartSize*). Note that constriction speed is not significantly different from HP for AMD patients, indicating that pupil constriction velocity remains in the normal range for these patients. Comparisons of AMD and DR patients indicate which PLR characteristics significantly differ between the two groups. The distributions of these variables are shown in Supplementary Figure 2 (Table 3).

**Table 3:** Statistics for the PLR variables of the right eye.

Variables PLR RE	HP n55 vs AMD n74		HP n55 vs DR n56		DR n56 vs AMD n74	
	*p*	Cohen’s d	*p*	Cohen’s d	*p*	Cohen’s d
Start Lat	0.299	−0.19	0.002	−0.61	0.014	−0.44
Min PSize	0.000	0.72	0.022	0.44	0.124	−0.27
Max Lat	0.026	−0.40	0.001	−0.63	0.232	−0.21
Start Size	0.018	−0.43	0.000	0.69	0.000	1.13
Min PSize	0.198	−0.23	0.046	0.38	0.001	0.59
P speed	0.197	0.23	0.000	−0.91	0.000	−1.21

Comparisons between HP and AMD, HP and DR, and between AMD and DR. *P* is Student’s *t*-test probability of a difference (*p* < 0.05 in green) and Cohen’s *d* is the associated effect size (values above 0.6 or below −0.6 in yellow).

#### Eye movements

Table 4 presents the statistics for these eye-movement variables. It can be seen that the fixation of AMD patients was significantly worse (higher standard deviations of eye position) than that of HP, but AMD patients did not make significantly more saccades away from fixation (number of outliers detected in the eye movement traces). In contrast, DR patients had similar eye position variability as compared to HP, but made significantly more saccades away from fixation. Comparisons of AMD and DR patients indicate the two groups differ with regard to eye-movements, although with medium effect sizes. The distributions of these variables are shown in Supplementary Figure 3.

**Table 4:** Same as Table 3 for eye movement variables.

Eye movement Variables RE	HP n55 vs AMD n74		HP n55 vs DR n 55		RD n55 vs AMD n74	
	*p*	Cohen’s d	*p*	Cohen’s d	*p*	Cohen’s d
Std Hor	0.01	−0.51	0.81	−0.05	0.02	0.46
Std Ver	0.00	−0.70	0.06	−0.38	0.04	0.39
Out Hor	0.12	−0.29	0.01	−0.53	0.14	−0.27
Out Ver	0.69	0.07	0.02	−0.45	0.00	−0.55

In column 1, ‘Std’ refers to the standard deviation of eye position, and ‘Out’ indicates the number of eye-positions farther away than 2 standard deviations of the mean (outliers). Cells in green indicate *p* < 0.05; cells in yellow indicate Cohen’s d effect size <-0.6 or >+0.6.

#### Pupil state during mPFT

Table 5 and Supplementary Figure 4 present the statistics for the global pupillary variables measured during mPFT (see Method). AMD patients had a smaller minimum pupil size than HP, but were similar to HP otherwise. All but one value measured for DR patients were significantly different from those of HP.

**Table 5:** Same as Table 4 for global Pupil State variables.

PUPIL variables RE	HP n 55 vs AMD n74		HP n55 vs DR n55		AMD n74 vs DR n55	
	*p*	Cohen’s d	*p*	Cohen’s d	*p*	Cohen’s d
Avg Pup	0.08	−0.32	0.05	0.38	0.00	0.68
Max Pup	0.71	−0.07	0.02	0.47	0.00	0.58
Min Pup	0.02	−0.43	0.12	0.31	0.00	0.71
Amp Pup	0.14	0.27	0.00	0.69	0.01	0.48
Std Pup	0.69	−0.07	0.00	0.74	0.00	0.97
Slope	0.94	−0.01	0.01	−0.51	0.01	−0.49
XCor	0.01	0.52	0.00	0.91	0.20	0.27

See the method section for a description of the global state variable. Cells in green indicate *p* < 0.05; cells in yellow indicate Cohen’s d effect size <-0.6 or >+0.6.

Likewise, AMD and DR patients also differed for most of these variables. Of note, the Xcor variable that estimates the lag between the stimulus and pupil modulations is significantly longer for the DR and AMD groups as relative to the HP group, indicating a slowing down of the processing time through the retino-pupillary circuits.

#### Oscillatory pupil response, spectral analyses

**Figure 2:**
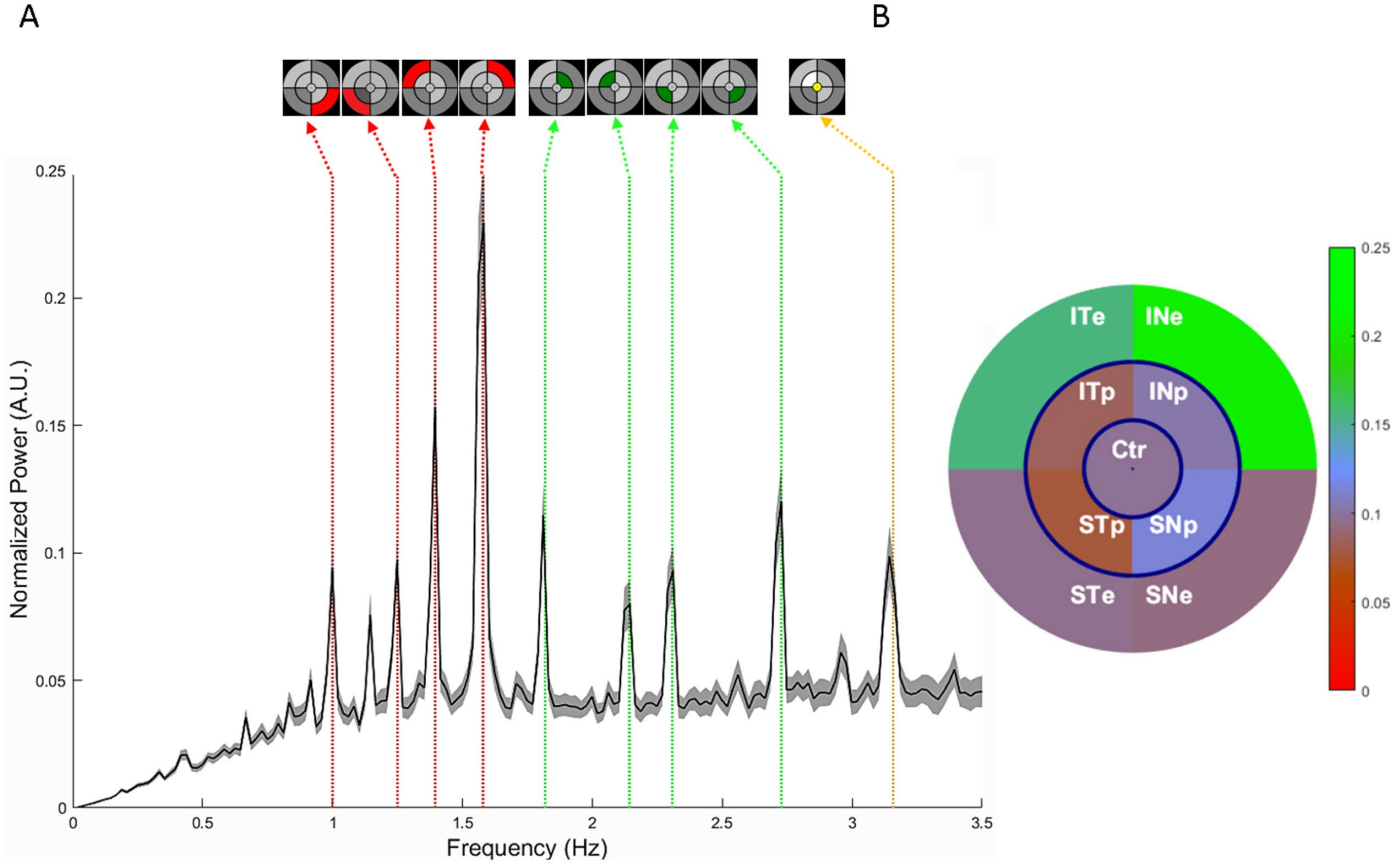
**(A)** Normalized mean spectral power between 0 and 3.5 Hz averaged over 63 right eyes of the HP group; 95% confidence intervals are shown in gray. A normalization was applied to the raw power spectrum by multiplying the power at each frequency by the square of the frequency, to compensate for the 1/*f*^2^ decrease of power. The vertical dotted lines indicate the frequencies of interest (FOIs: red for peripheral sectors, green for paracentral sectors, and yellow for the central sector). The arrows show the correspondence between FOIs and the 9 sectors of the mPFT stimulus (top). **(B)** Regional distributions of the power extracted from the pupillary responses plotted onto a multifocal pupillary response field. Regions are labeled with their retinal projection. The color bar on the right indicates the spectral power in arbitrary units.

The spectral power extracted for each FOI during mPFT permit mapping multifocal Pupillary Response Fields (mPRF, Figure 2) showing the retinal sectors with lower or higher spectral power. As it can be seen in Table 6, the FOI power is significantly less for several sectors in both the AMD and DR groups as compared to HP (also see Supplementary Figures 5, 6). Of interest is that the power at the central and some paracentral sectors are significantly less in AMD with large effect sizes for some sectors, in line with the retinal damage characteristic of this pathology. For DR patients, all, except one, sectors are showing reduced power, in line with the diffuse alterations of the retina due to this pathology. The FOI power is significantly different in some sectors for the AMD and DR groups, indicating distinctive patterns of mPRF between these patients, although the effect sizes are small.

**Table 6:** Same as Table 4 for each FOI power.

Variables FOI power RE	HP n55 vs AMD n74		HP n55 vs DR n56		RD n56 vs AMD n74	
	*p*	Cohen’s d	*p*	Cohen’s d	*p*	Cohen’s d
F1	0.80	0.04	0.05	0.38	0.11	0.28
F2	0.00	0.70	0.00	0.83	0.40	0.15
F3	0.00	0.55	0.00	0.86	0.11	0.29
F4	0.28	0.20	0.00	0.83	0.00	0.59
F5	0.02	0.43	0.00	0.65	0.21	0.22
F6	0.34	0.17	0.00	0.78	0.01	0.48
F7	0.00	0.72	0.00	0.72	0.95	0.01
F8	0.00	0.60	0.00	0.83	0.22	0.22
F9	0.00	0.80	0.01	0.54	0.12	−0.28

F1–F9 stands for the 9 sectors/frequencies. Cells in green indicate *p* < 0.05; cells in yellow indicate Cohen’s d effect size <-0.6 or >+0.6.

Figure 2A represents the average mPRF of the HP group. Note the large spectral power of the eccentric sector projecting onto the infero-nasal retina (INe) which indicates that this region entail greater pupillary responses than other sectors, in agreement with previous reports (Schmid et al., 2000; Wilhelm et al., 2000). This finding is in line with the higher density of ipRGCs found in the infero-nasal retina, (Hannibal et al., 2017) and demonstrates that mPFT reflects the retinal distribution of these cells. A symmetrical pattern is observed for the left eye.

#### Relative phase between stimulus and pupil

Phase lags between the stimulus and pupil oscillations are key features for assessing pupillary responses across regions in different clinical conditions and inform on a slowing down of processing in specific disease-dependent regions. These lags could result from a smaller number of cells contributing to the response, causing lengthened integration time, or to a focal malfunction of a damaged retinal circuitry.

**Figure 3:**
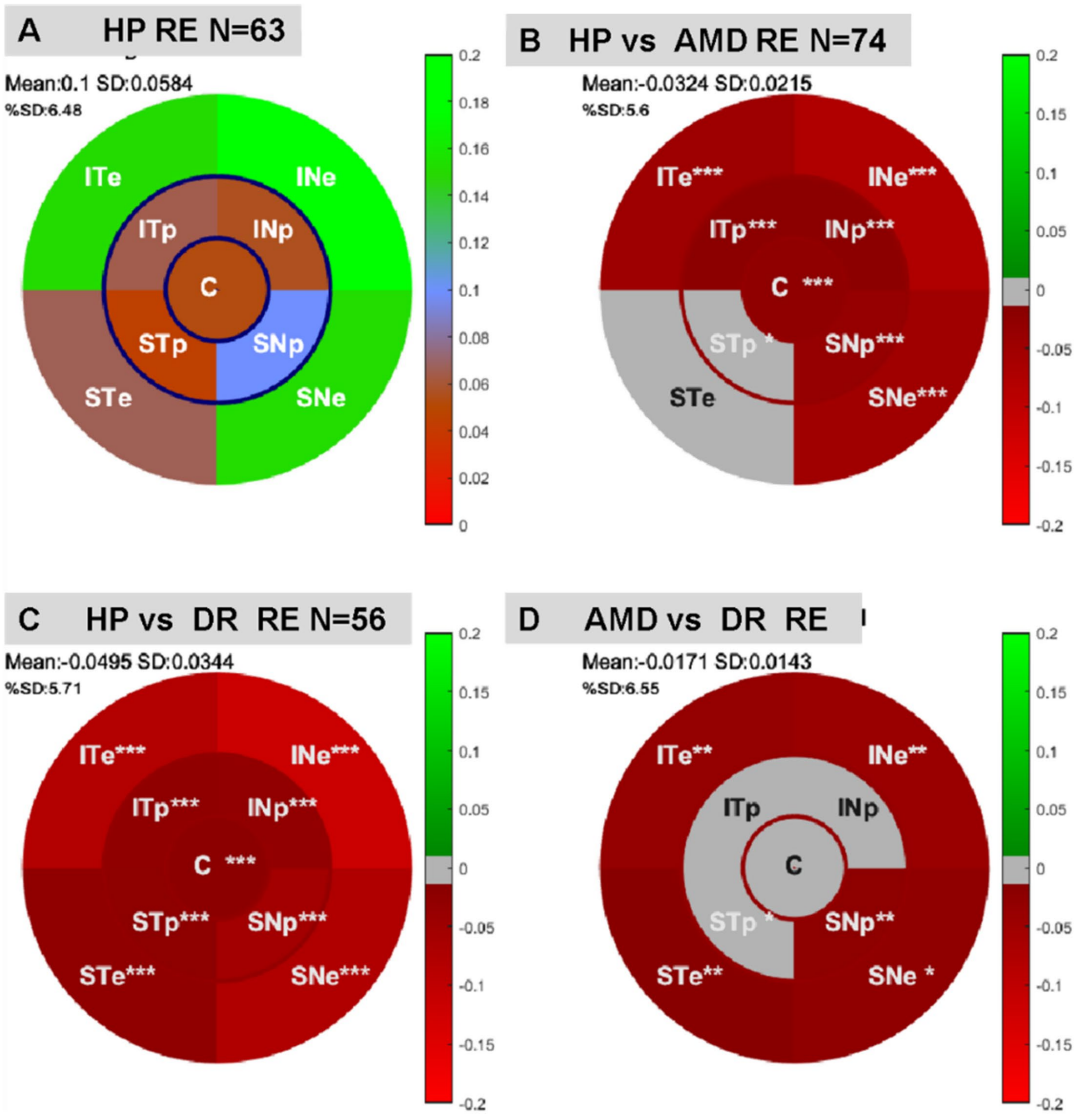
**(A)** Average mPRF of the right eye for the HP group. (B, C) Regional distributions of statistical differences of multifocal pupillary response field between HP and patients or the right eye. **(B)** Differences between the mPRF of the HP and DR groups. **(C)** Differences between the mPRF of the HP and AMD groups. **(D)** Differences between the mPRF of the DR and AMD groups. *p* < 0.05 *, *p* < 0.01 **, *p* < 0.001 ***.

We used circular statistics routines (Berens, 2009) to determine whether the distribution of phase lags was significantly different between groups. This was done without removing outliers given the peculiar distribution of this circular variable. Table 7 summarizes the results and polar plots of phase lag distributions are shown in Figure 3.

**Table 7:** Results for each FOI phase (P1 to P) computed with circular statistics.

PHASE variables RE	HP n 55 vs AMD n 74	HP n 55 vs DR n 55	AMD n74 vs DR n55
	*p*	*p*	*p*
P1	0.00	0.00	0.00
P2	0.00	0.00	0.00
P3	0.00	0.00	0.00
P4	0.01	0.00	0.00
P5	0.97	0.76	0.00
P6	0.01	0.15	0.00
P7	0.00	0.00	0.00
P8	0.24	0.15	0.00
P9	0.00	0.00	0.00

Effect size were not computed for phases. Cells in green indicate *p* < 0.05; cells in yellow indicate Cohen’s d effect size <-0.6 or >+0.6.

As it can be seen in Table 7, phase lags are significantly different for 7 out of 9 sectors for AMD patients and for 6 out of 9 sectors for DR. The distributions of phase lags in Figure 3 indicate that pupillary responses are delayed in AMD and DR relative to the HP group, differentially for different regions. Note the large and consistent differences between HP and patients for the central sector (Figure 3I).

**Figure 4:**
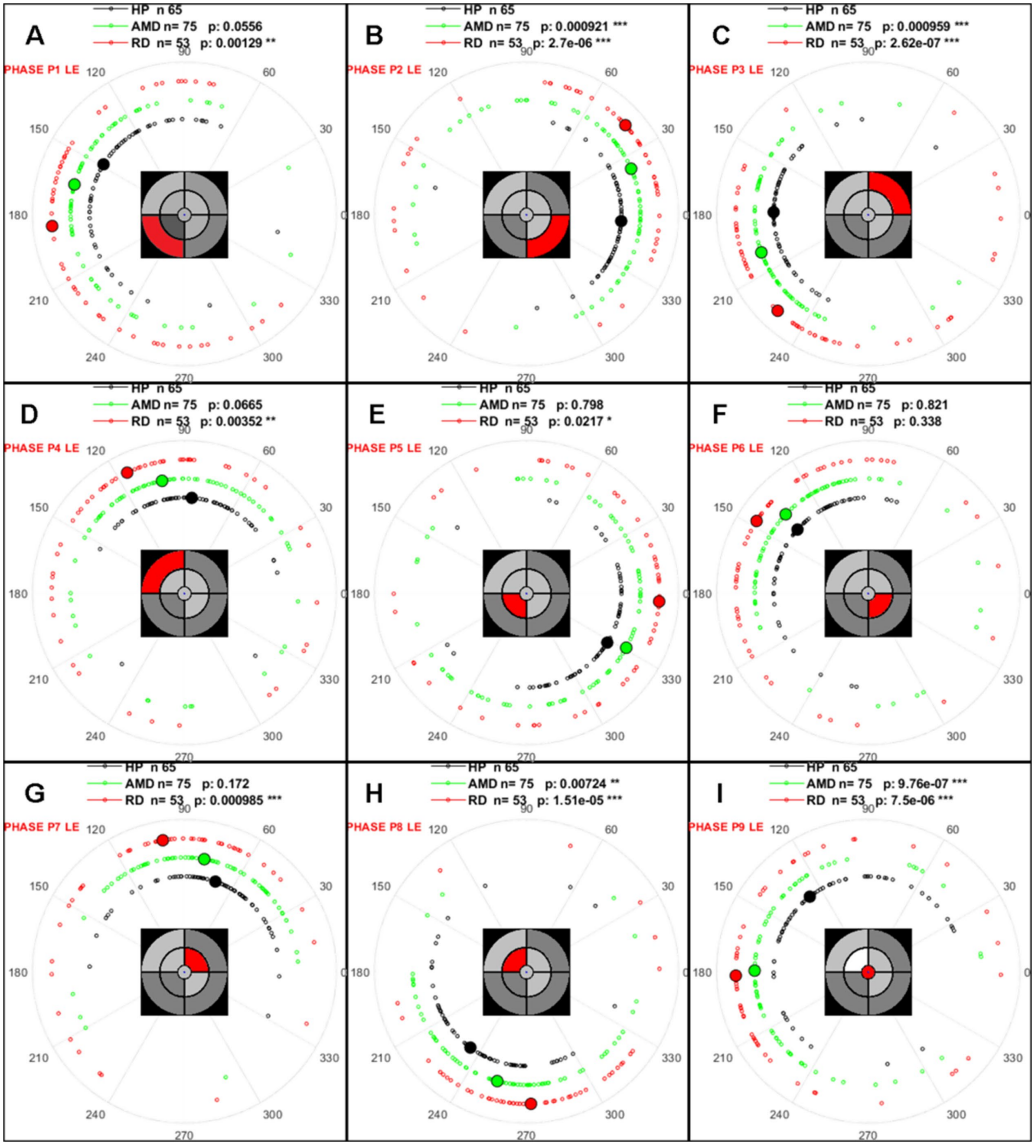
Distributions of phase lags for each of the 9 sectors for the different groups. **(A–I)** Small open symbols are individual data; large filled symbols represent the circular mean of each group. HP, black; DR, red; AMD, green. The insets indicate the statistical significance of the differences between the HP and the patient groups for each sector shown in the insets (also see Table 7).

Two observations deserve comments. First, the spread of phase lags is larger for the patient groups than for HP, suggesting large inter-individual differences within the AMD and DR groups. This observation could relate to the severity, diversity and spatial distribution of retinal damage. Secondly, the phase lags differ between sectors. This is because phase lag values depend upon the frequency coupled to each sector, being a fraction of the associated periods. Converting phase lags into time delays is not trivial because phase lags are computed modulo *2kpi*. Calculating delays from phase lags by adjusting *k* requires making assumptions on plausible delays. This is not obvious as processing delays can have many origins, including the integration time from photoreceptors to ganglion cells, possibly within damaged circuits, the propagation along the retina through unmyelinated fibers, whose timing depends on the distance to the optic disk, and possibly on the integrity of the retinal nerve fiber layer, or to propagation through the optic nerve and within pupillary circuits. We preferred not to make such assumptions and kept phases as a measure of lags, as we were interested in comparing HP and patients for each sector independently (no comparisons of relative lags between sectors). Note that phase shifts across sectors are consistent across groups suggesting that they reflect a veridical feature of the pupil response. It is however legitimate to convert the differences of phase lags for each frequency/sector and to evaluate the corresponding time delay differences for each sector between groups. Figure 4 shows the mean phases, the differences of mean phases, and the corresponding delays by sector/frequency between HP and patients.

**Figure 5:**
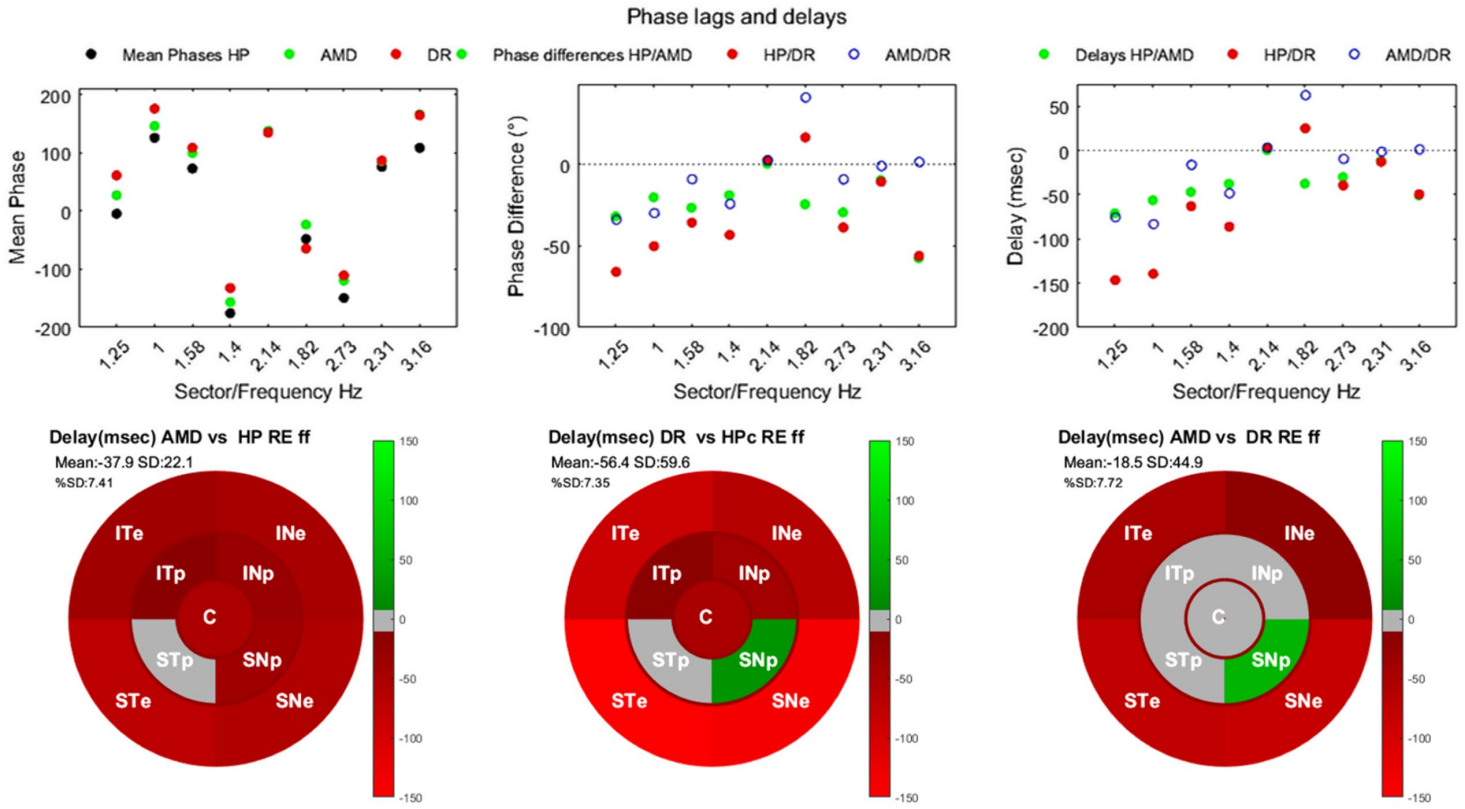
Phase lags and delays for the right eye. **(A)** Mean phase lags for each group and each sector/frequency. HP, black symbols; AMD, green symbols; DR, red symbols. **(B)** Differences of mean phase lags by sector/frequency. Filled symbols shows the differences between HP and patients groups. Open symbols show the differences between AMD and DR. **(C)** Time delays between the HP and patients. Filled symbols shows the differences between HP and patients groups. Open symbols show the differences between AMD and DR. **(D–F)** Maps of delays for AMD vs. HP, DR vs. HP, and AMD vs. DR, respectively. The figure shows the computed relative power ratios.

The calculated delays in Figure 4C depend on sectors but range between −73 and −14 msec. For AMD relative to HP, and between −110 and + 22 msec. For DR relative to HP (Figures 4D,E). In addition, the delays between AMD and DR range from −73 to +62 msec. These results indicate that regional processing delays measured with mPFT provide insight into the impact of disease on response timing and inform on the physiopathology and functional consequences of diseases. The delays observed herein are larger than those reported with the multifocal electroretinograms (mfERG) or multifocal Visual Evoked Potential (mfVEP) methods (Li, 2001; Hood et al., 2006; Ng et al., 2008). This is expected as both methods bypass the more sluggish pupil circuits while mPFT relies on pupil responses occurring after recruiting the whole retino-pupillary circuits. Nevertheless, the present results point to a similar slowing down of processing in pathologies, supporting the view that delays between stimulus and response are relevant biomarkers of ophthalmic diseases (Figure 5).

#### Relative power between regions

Ophthalmic diseases can lead to intraocular asymmetrical damage between retinal regions. Analyzing relative power between mPFT sectors could therefore bring additional information to characterize pathologies. We investigated whether asymmetries exist in pupil responses by computing different regional spectral power ratios of up/down and left/right quadrants and hemifields, between paracentral and eccentric sectors, and central versus all other sectors.

Table 8 shows the statistics for the different relative power ratios. Strikingly, none of the up/down ratios showed significant differences between HP and patient groups, while all left/right ratios were significantly different in patients as compared to HP. In addition, the ratio of the power of the center sector differed significantly in AMD patients, but not in DR patients. Moreover, the AMD and DR groups significantly differ for that ratio.

**Table 8:** Statistics comparing relative power ratios in HP and patient groups.

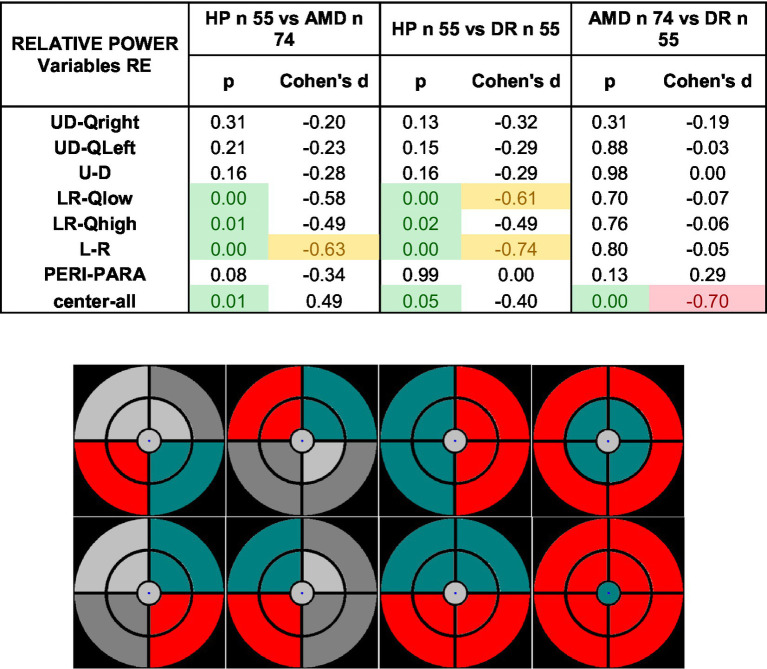

These differences indicate that anisotropic spectral power characterizes AMD and DR, and further strengthen the view that mPFT does characterize disease-specific features (also see Supplementary Figure 8).

### AUC of ROC

AUCs of ROC were calculated using various combinations of the variables described above to determine whether they differentiate the HP and patient groups. We report here the AUC, sensitivity and specificity obtained using: FOI power alone, FOI power combined with phase, and FOI power combined with global pupil state and relative power variables. AUCs of ROC were also computed using only the PLR features for comparison. These analyses were conducted for AMD, DR or both groups, with the right or left eye or with both eyes. We also performed ROC analyses on datasets including or excluding outliers, either including all participants or subsets with low rates of blinks and transients.

Table 9 shows some of these analyses for each patient group or for both AMD and DR. Only right-eye results are presented in this table. A table with all the results is available in Supplementary Table 3.

**Table 9:** Results of AUCs of ROC for the right eye, computed with different combinations of features described in column 1.

AUC of ROC	Right EYE				
A	AUC	CI95 low	CI95 high	Sensitivity	Specificity
Variables for ROC	HP n 55 vs AMD n 74				
FOI_9_ Power	0.85	0.75	0.90	0.90	0.64
FOI_9_ Power, Pupil_5_, REL_4_ Power	0.93	0.85	0.97	0.95	0.83
FOI_9_ Power Phases	**1**	**1**	**1**	**1**	**1**
FOI_9_ Power Phases, Pupil_5_, REL_4_ Power	1	1	1	1	1
PLR_6_	0.91	0.82	0.95	0.94	0.77
B	HP n 55 vs DR n 56				
FOI_9_ Power	0.85	0.76	0.91	0.96	0.57
FOI_9_ Power, Pupil_5_, REL_4_ Power	0.96	0.91	0.96	0.87	0.93
FOI_9_ Power Phases	1	1	1	1	1
FOI_9_ Power Phases, Pupil_5_, REL_4_ Power	**1**	**1**	**1**	**1**	**1**
PLR_6_	0.83	0.73	0.89	0.68	0.87
C	HP n 55 vs AMD 74 + DR n 56				
FOI_9_ Power	0.81	0.75	0.88	0.96	0.43
FOI_9_ Power, Pupil_5_, REL_4_ Power	0.92	0.85	0.94	0.94	0.76
FOI_9_ Power Phases	1	1	1	1	1
FOI_9_ Power Phases, Pupil_5_, REL_4_ Power	**1**	**1**	**1**	**1**	**1**
PLR_6_	0.76	0.65	0.84	0.87	0.5
D	AMD 74 vs DR n 56				
FOI_9_ Power Phases	0.91	0.8	0.96	0.86	0.84
FOI_9_ Power, REL_4_ Power	0.95	0.88	0.99	1	0.81
FOI_9_ Power Phases, REL_4_ Power	**1**	**1**	**1**	**1**	**1**
PLR_6_	0.77	0.68	0.84	0.9	0.51

Columns 2–4 are the AUCs and corresponding 95% confidence intervals. Columns 5–6 are the sensitivity and specificity. Best results are shown in bold. A, HP versus AMD; B, HP versus DR. C, HP versus AMD + RD. D, AMD vs. DR.

As it can be seen, the AUC for classifying HP vs. AMD, HP vs. DR or HP vs. any pathology, measured with only FOI power is around 0.8 for all classifications. AUCs of 1 are obtained for AMD and DR when FOI power and phase are used, or when other variables are added. Specificity is high when considering AMD or DR separately, but less when both groups are merged. PLR-based classifications yielded lower performance, though AUCs remained above 0.9 for AMD and 0.8 for DR, but sensitivity or specificity are less in these cases. AUCs of 1 were obtained when using Power, Phases and Relative power to classify AMD and DR.

Overall, the results show that the variables derived from the analyses of mPFT enable discrimination between the different groups with excellent sensitivities and specificities.

### Other factors potentially influencing pupil spectral power

#### Cataract

Some participants were diagnosed with a cataract, and some had undergone posterior-chamber intraocular lens (PCIOL) implantation. A cataract could diminish the quantity of light entering the eye and hence modulate pupil reactivity. To evaluate whether these different conditions have an effect on pupil responses we compared the distributions of the mean spectral power for different cataract severity levels (Student’s t-tests). Cataract severity was graded as follows: CAT0 (no cataract, *N* = 53), CAT1 (moderate, *N* = 38), CAT2 (severe, *N* = 30), PCIOL was noted −1 (*N* = 89). These comparisons were performed both across all participants and within each diagnostic group. No significant differences were found, except for DR for which the power differed for CAT0 and CAT2 conditions, although only limited data (*N* = 7) were available in that condition (Supplementary Figure 9).

#### Diseases’ characteristics

AMD patients presented different forms of AMD: Geographic atrophy (*N* = 14), Neovascular AMD (*N* = 60) and early or intermediate AMD (*N* = 18). Student’s t-tests were performed to determine whether the mean spectral power differed across these subgroups. No significant differences were found (Supplementary Figures 10a,b).

Two forms of DR were present in the DR group: non-proliferative DR (NPDR, *N* = 24) or proliferative DR (PDR, *N* = 32). Student’s t-tests were performed to determine whether the mean spectral power differed between these subgroups. No significant difference was found between the NPDR and PDR subgroups (Supplementary Figures 10c,d).

#### Visual acuity

Visual acuity varies between subjects and could modulate the spectral power during mPFT, despite the use of large stimulus sectors. In addition, because HP were only included if their visual acuity was equal to, or better than 8/10, HP differed significantly from the patient groups in this regard. To evaluate whether visual acuity was a potential confounding factor, we calculated Pearson’s correlation coefficients to characterize the relationship between averaged spectral power and visual acuity (Supplementary Figure 11). When the data from all groups were pooled, we found a significant correlation between visual acuity and mean spectral power (*r* = 0.29; *p* < 0.001), as well as a significant correlation between age and visual acuity (*r* = −0.36; *p* < 0.001). In contrast, when each group was analyzed separately, no significant correlations were found for the HP (*r* = 0.11; *p* = 0.42), AMD (*r* = 0.19; *p* = 0.097), or DR (*r* = 0.21, *p* = 13) groups. However, when considering only the spectral power in the central sector, rather than the mean spectral power across all sectors, we did find a significant correlation for AMD patients (*r* = 0.37, *p* < 0.005), but not for the HP (*r* = −0.02, *p* = 0.88) or DR (*r* = 0.08, *p* = 0.53) groups.

Significant age-visual acuity correlations were also observed for the HP group (*r* = −0.66; *p* < 0.01) and for the pooled AMD and DR data (*r* = −0.36; *p* < 0.01). These analyses suggest that spectral power increases slightly with increasing visual acuity, but that this effect is mostly related to age.

## Discussion

The results indicate that pupil responses and eye movements recorded during a one-minute test requiring only passive fixation can generate multifocal Pupil response Fields that reflect retinal damage patterns characteristic of AMD and DR. Derived variables reliably distinguish between HP, AMD and DR patients with high sensitivity and specificity. These findings are robust across different data corrections and selection criteria, including exclusion of trials with excessive artifacts, outlier removal, and age correction. This study builds on our previous work involving other pathologies such as glaucoma, (Stelandre et al., 2023), retinitis pigmentosa, Stargardt disease, and Leber’s hereditary optic neuropathy (Ajasse et al., 2022; Lorenceau et al., 2024).

These new results are consistent with prior studies using different techniques (mfERG, mfVEP), which also report processing delays in AMD or DR. They align with pupillography studies showing reduced pupil response amplitudes in various diseases. Finally, the mPRFs obtained with our method are consistent with anatomical studies, such as those reporting higher density of ipRGCs in the infero-nasal retina (Hannibal et al., 2017). This anatomical-functional correspondence reinforces the physiological relevance of mPFT. The strong spectral power observed in the infero-nasal sector in healthy participants likely reflects the topographic distribution of ipRGCs, and supports the view that mPFT can capture spatial variations in the integrity of retino-pupillary pathways. Such physiopathological specificity strengthens the interpretation of mPFT signals as meaningful markers of regional retinal dysfunction.

Several methodological considerations warrant further discussion.

The mPFT method uses 9 temporally modulated luminance frequencies (TMFs), each coupled to a particular region. One may question whether these frequencies alone could account for the observed spectral power differences between sectors. This issue was previously investigated (Lorenceau et al., 2024) using alternative TMF/Sector couplings. The finding demonstrated that spectral power distributions reflect underlying retinal properties rather than being driven by TMFs alone. Supporting this conclusion, studies investigating the influence of TMFs on pupil responses (Clarke et al., 2003; Barrionuevo et al., 2023) have shown minimal variation in pupil response amplitude within the 1–4 Hz range, with a marked decline above 4 Hz. The highest TMF used here (3.15 Hz) was assigned to the central disk, an intentional choice made to mitigate the otherwise dominant contribution of the macula to overall pupil response (Hong et al., 2001).

Another potential concern is whether dark adaptation is required before mPFT testing. As noted in the introduction, dark adaptation for 5–10 min is impractical in routine clinical settings. Our previous work demonstrated that it is not necessary: mPFT relies on a sustained, global retinal drive that effectively engages the entire retina without prior adaptation. Comparing mPFT results obtained after daylight versus dark adaptation revealed no significant differences in the *relative* distribution across retinal regions (Lorenceau et al., 2024). Because mPFT induces inter-regional competition, it has the potential to reflect focal retinal damage relative to other regions with little dependence on the adapted state or on more global factors (fatigue, drugs, or systemic treatments) that may affect overall pupillary responses, but not of the relative contribution of each sector compared to others.

A further consideration concerns the potential influence of attention, whether directed toward specific region or acting a non-specific modulator of overall pupil responses. Our previous study also addressed this point and showed no significant effect, at least for large luminance modulations where the visual mPFT drive is strong and overcomes attentional effects. As a matter of fact, attentional effects or those of other cognitive factors (e.g., memory load) through sympathetic activation are small and unable to balance the strong parasympathetic drive of the retino-pupillary circuits induced by mPFT. Test–retest reliability was also evaluated in a previous study, yielding a strong inter-trial correlation (*r* = 0.82).

### Limitations

The present study has several limitations. First, included patients are already diagnosed with AMD and DR, sometimes at an advanced stage. In this regard, the study was primarily designed to demonstrate that mPFT provided results comparable to the known impacts of AMD and DR diseases on vision, while adding novel information. It was not within the scope of this study to expand enrollment to a large population with a sufficient number of patients at different stage of their disease to evaluate whether mPRFs scale with disease severity. Additional work is needed to test whether mPFT can be used to screen for early pupillary biomarkers that would signal the onset of a disease at an early stage. Also, assessing the potential effects of other factors (Iris color, intraocular pressure, previous surgery, etc.), would help refine the analyses.

The gray mPFT version used in this study involves high-contrast luminance modulations, which limits its capacity to disentangle the respective contribution of rods and cones, as these photoreceptors may be differentially affected in AMD and DR. Tailoring the mPFT protocol, by reducing luminance contrast or incorporating chromatic stimuli, could enhance its sensitivity to early dysfunction and help identify photoreceptor-specific deficits. It would therefore be relevant to explore chromatic adaptations of the mPFT protocol, an avenue we are currently investigating and which will be addressed in the future.

Finally, the mPFT sectors used here are large, and thus unable to uncover a focal retinal defect, contrary to mfERG for instance. However, mfERG often requires pooling data across sectors to improve signal to noise ratio, which also compromises spatial precision. It is also worth noting that the diseases considered here rarely entrain a single focal dysfunction. Moreover, a focal damage covered by a sector may still modulate the pupil response, although its precise localization would remain elusive, and the effect would be reduced as compared to more extended defects. We already tested mPFT versions with smaller sectors located in different quadrants (Ajasse et al., 2022), that permit more focal assessments, although these additional tests lengthen the pupillary examination.

### Future directions

Although this and previous studies (Ajasse et al., 2022; Stelandre et al., 2023; Lorenceau et al., 2024) confirm the interest of mPFT for clinical assessment, it is not yet routinely used in the clinic, or for screening individuals at risk for developing ophthalmic diseases. Longitudinal studies are needed to evaluate whether mPFT has the potential to detect a developing disease before vision is compromised, or whether it can predict the furture occurrence of a disease, not detected with structural examination with retinal imaging (OCT for instance).

Another line of research concerns the relationships between structural deficits and pupil responses, as well as a better understanding of the origins of reduced pupil power in some regions (e.g., some paracentral sectors in AMD) to determine whether they correspond to a compromised structural substratum. Further testing mPFT in other –ophthalmic or neurologic- pathologies would allow determining whether mPFT generalizes to a wide range of diseases. Finally, it would be interesting to determine whether mPFT can be used to probe the efficacy of treatments such as drugs or gene therapy under development.

## Conclusion

Multifocal Pupillary Response Fields (mPRFs), as assessed in the study, offer valuable and multifaceted insights into visual function in patients with neuropathies or retinopathies, including regional estimates of slowed visual processing.

The mPFT method is rapid, objective, non-invasive, does not require specialized expertise, and is independent of subjective responses. The test is well accepted by patients (see questionnaire summary in Supplementary Figure 12), and has already demonstrated efficacy across several pathologies (Stelandre et al., 2023; Lorenceau et al., 2024), positioning mPFT as a novel, easy-to-use tool for the functional evaluation of vision. The present results enable partial mapping of retinal damage in AMD and DR, and support accurate classification of patients versus healthy controls, as well as differentiation between AMD and DR cases. The extent to which these findings will contribute to a better understanding of the pathophysiology of these diseases requires further investigation.

Moreover, since the pupil regulates the amount of light entering the eye, evaluating the integrity of the retino-pupillary circuits offers intrinsic diagnostic value not captured by standard visual tests. This may help identify the origin of symptoms such as photophobia, glare sensitivity, or even migraine in certain patients. This approach holds significant potential for clinical implementation, not only as a diagnostic tool, but also for longitudinal monitoring and therapeutic evaluation across retinal and neuro-ophthalmologic conditions.

## Data Availability

The original contributions presented in the study are included in the article/Supplementary material, further inquiries can be directed to the corresponding author.
