# Supplementary material for: Frequency tagged multifocal pupillary response fields identify age-related macular degeneration and diabetic retinopathy
Source: Front Neurosci. 2026 Feb 10;19:1653938. doi: 10.3389/fnins.2025.1653938 (PMC12929433; doi:10.3389/fnins.2025.1653938)
Supplement: Supplementary file 2 [file Data_Sheet_1.docx]

**Frequency tagged Multifocal Pupillary Response Fields identify age-related macular degeneration (AMD) and diabetic retinopathy (DR)**

Laure Trinquet, Thierry David, Fredéric Chavane, Jean Lorenceau, Frederic Matonti

**Supplementary Text**

English translation of the questionnaire given to the participants before and after the mPFT test:

**IRIDYN Study**

Date:
Participant Inclusion Number:

**Section 1 – Beginning of Session**

*Read and completed by the examiner*

General Fatigue

How would you rate your current level of fatigue?
Not tired
1 ☐ 2 ☐ 3 ☐ 4 ☐ 5 ☐ 6 ☐ 7 ☐ 8 ☐ 9 ☐ 10 ☐ Very tired

Mood

How do you feel emotionally at the moment?
Very poor
1 ☐ 2 ☐ 3 ☐ 4 ☐ 5 ☐ 6 ☐ 7 ☐ 8 ☐ 9 ☐ 10 ☐ Very good

Ocular Fatigue

Do you currently experience any eye fatigue (e.g., pulling, burning, tearing, stinging…)?
Not at all
1 ☐ 2 ☐ 3 ☐ 4 ☐ 5 ☐ 6 ☐ 7 ☐ 8 ☐ 9 ☐ 10 ☐ Yes, very much

Medications

Are you currently taking any medication?
Yes / No
If yes, which one(s)? ______________________________________

Visual Field Testing History

Have you ever had an automated or Goldmann visual field test?
Yes ☐ No ☐ I don’t know ☐

Rate your experience with visual field testing (0–10):
1 ☐ 2 ☐ 3 ☐ 4 ☐ 5 ☐ 6 ☐ 7 ☐ 8 ☐ 9 ☐ 10 ☐

**Section 2 – End of Session**

*Read and completed by the examiner*

Session Duration

How did you find the duration of the session?
Acceptable
1 ☐ 2 ☐ 3 ☐ 4 ☐ 5 ☐ 6 ☐ 7 ☐ 8 ☐ 9 ☐ 10 ☐ Much too long

Glare Discomfort

Did you feel dazzled or bothered by the brightness of the tests?
Not at all
1 ☐ 2 ☐ 3 ☐ 4 ☐ 5 ☐ 6 ☐ 7 ☐ 8 ☐ 9 ☐ 10 ☐ Yes, very much

Fixation Difficulty

Did you have difficulty fixating on the targets?
Not at all
1 ☐ 2 ☐ 3 ☐ 4 ☐ 5 ☐ 6 ☐ 7 ☐ 8 ☐ 9 ☐ 10 ☐ Yes, very much

Fixation Target Size

In your opinion, were the fixation targets too small?
Yes / No

Fixation Target Contrast

In your opinion, were the fixation targets insufficiently contrasted?
Yes / No

Test Brightness

Do you feel that the test was too dazzling?
Yes / No

Postural Comfort

Was the posture uncomfortable (chair, chinrest, etc.)?
Yes / No

Ocular Fatigue After the Session

Do you feel more eye fatigue now than at the beginning (pulling, burning, tearing, stinging…)?
Not at all
1 ☐ 2 ☐ 3 ☐ 4 ☐ 5 ☐ 6 ☐ 7 ☐ 8 ☐ 9 ☐ 10 ☐ Yes, very much

General Fatigue After the Session

How would you rate your fatigue after the session?
Not tired
1 ☐ 2 ☐ 3 ☐ 4 ☐ 5 ☐ 6 ☐ 7 ☐ 8 ☐ 9 ☐ 10 ☐ Very tired

Global Rating – Pupillometry

Please give a global rating for this examination:
1 ☐ 2 ☐ 3 ☐ 4 ☐ 5 ☐ 6 ☐ 7 ☐ 8 ☐ 9 ☐ 10 ☐

Comparison with Visual Field Testing

Do you find this test easier to perform than a visual field test?
Yes ☐ No ☐ Similar ☐ I don’t know ☐

Why? _______________________________________________________

If you had to choose in the future between a visual field test and pupillometry, which would you prefer?
Visual field ☐ Pupillometry ☐ I don’t know ☐

General Comments

(Aspects to improve, general impressions, etc.)

**Supplementary Tables**

Supplementary Table 1: Data Correction. Statistics for the variables derived from the Data Correction procedure of the right eye. Comparisons between HP and AMD, HP and DR, and between AMD and DR. p is Student’s t-test probability of a difference (p<0.05 in green) and Cohen’s d is the associated effect size (values above 0.6 or below -0.6 in yellow). AMD patients made significantly more blinks than HP during the mPFT stimulus, while DR were not different in this regard. The number of data corrected for spurious transients shows the reverse, with RD presenting more transients than HP or AMD. AMD were comparable to HP for this variable.

*Distribution of ages in the different groups*

AMD and DR patients often are elderly individuals, generating a bias in the recruitment (see Table1). Recruiting healthy participants age-matched to patients is therefore uneasy, as individuals over 70 years often present health issues preventing their inclusion in the study. As a result, the distributions of age in the HP, AMD and RD groups were significantly different. To deal with this issue, we took advantage of the fact that the ages of HP span a large range (20-72 yo) to evaluate the effect of age on each variable, and to correct for this effect, when necessary. To that aim, a linear model was first fitted to the distributions of HP subjects with age as a regressor. Whenever the estimated slope of the fit was significant (p<0.05), the data of all participants were corrected using the fitted slope for the HP group so as to suppress the corresponding age-related trend for that variable. The effects of neuropathies on pupillary features could then be assessed after correcting, or not, the trend due to aging. Both possibilities were considered here to determine the impact of age on the statistical outcomes of the analyses. Results with and without correction were compared. We also performed the same analysis after removing all participants under 35 years old, to verify our conclusions would be robust, and not merely due to age.

Supplementary Table 2: Effects of age for HP participants and all participants for a subset of the variables of interest (Pearson’s correlation). Significant (p<0.05) values are shown in green. Only 4 variables were age dependent for the HP group, 3 of which were related to the PLR. Considering all participants, only 3 variables show a significant effect of age.

Supplementary table 3: AUC of ROC computed with different sets of variables. Results for the right and left eye and for both left and right eyes. The left most column indicate the variables that were used to compute AUC of ROC : FOI Power, FOI Phase, Pupil State variables, Variables derived from PLR analysis, Data corrections (Blinks and data corrected for spurious Pupil transients; Relative Power using only Left/Right, Up/Down, center/all Power ratios. Best AUC of ROC with fewer variables are in bold.

**Supplementary Figures**

Figures 1-8 below show the sorted distributions of each variable analyzed for each group, together with the statistical significance of the differences between the HP (black symbols) and the patient groups (AMD, green symbols; DR, red symbols). The statistics of the differences between DR and AMD patients are also shown. p is the probability of a significant difference and d is the effect size (Cohen's d) for each comparison. Stars: * p<0.05; ** p<0.01; *** p<0.001.


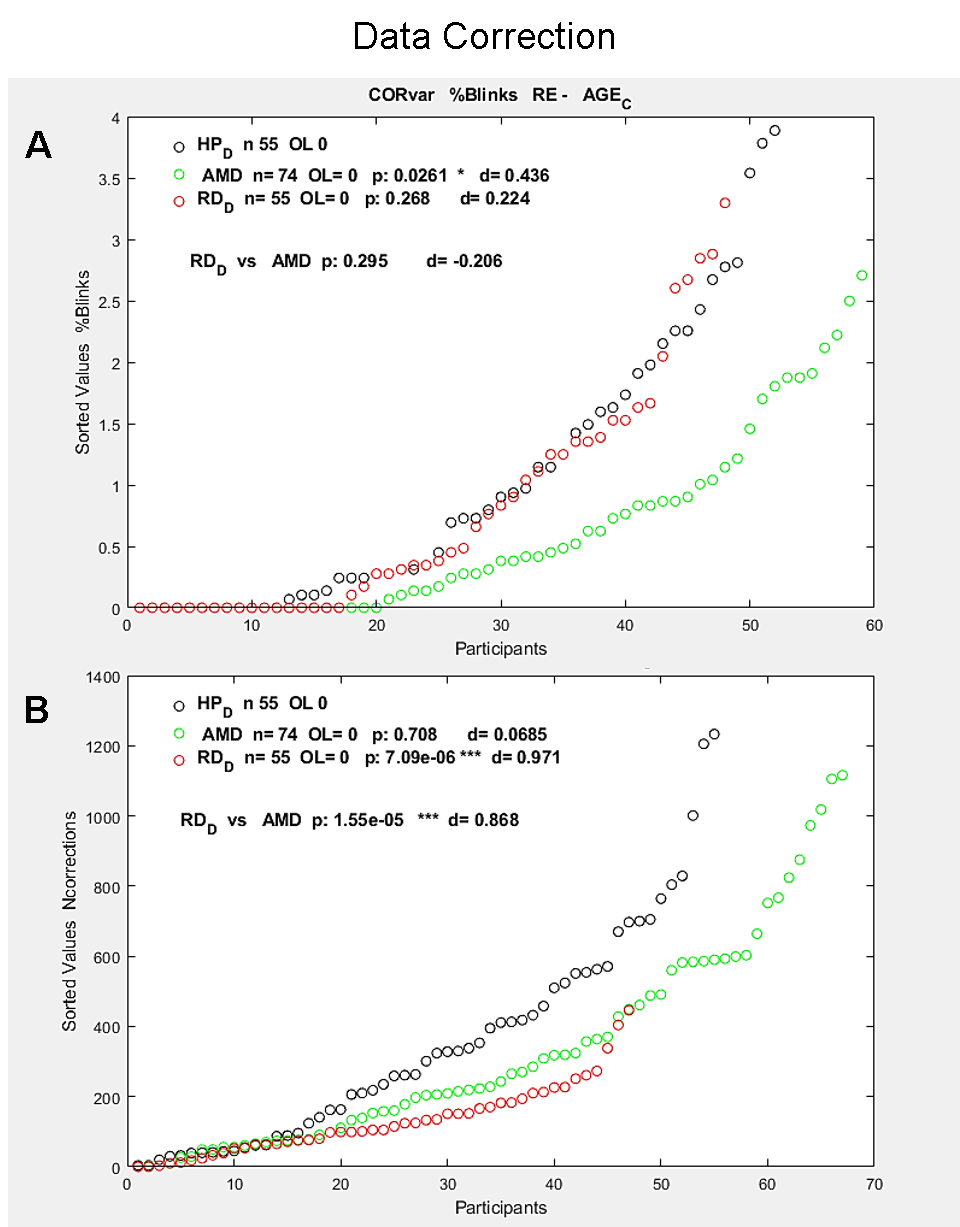


Supplementary Figure 1: Sorted distributions of data correction variables. A. Percentage of data points corrected for blinks. Blinks were detected as zero in the pupil traces. A pre- and post-blink of 4 frames (66 milliseconds) was used to replace blinks with smoothed linear interpolations. As can be seen, AMD and MLA patients had significantly more blinks than HP. B. Number of data points corrected for transients detected from the velocity of pupil changes, using a threshold to correct for fast pupil changes incompatible with the sluggish dynamics of the pupil. The detected values were replaced by a smooth linear interpolation between points. Significant differences were found between DR and HP compared to HP, and DR and MLA participants.


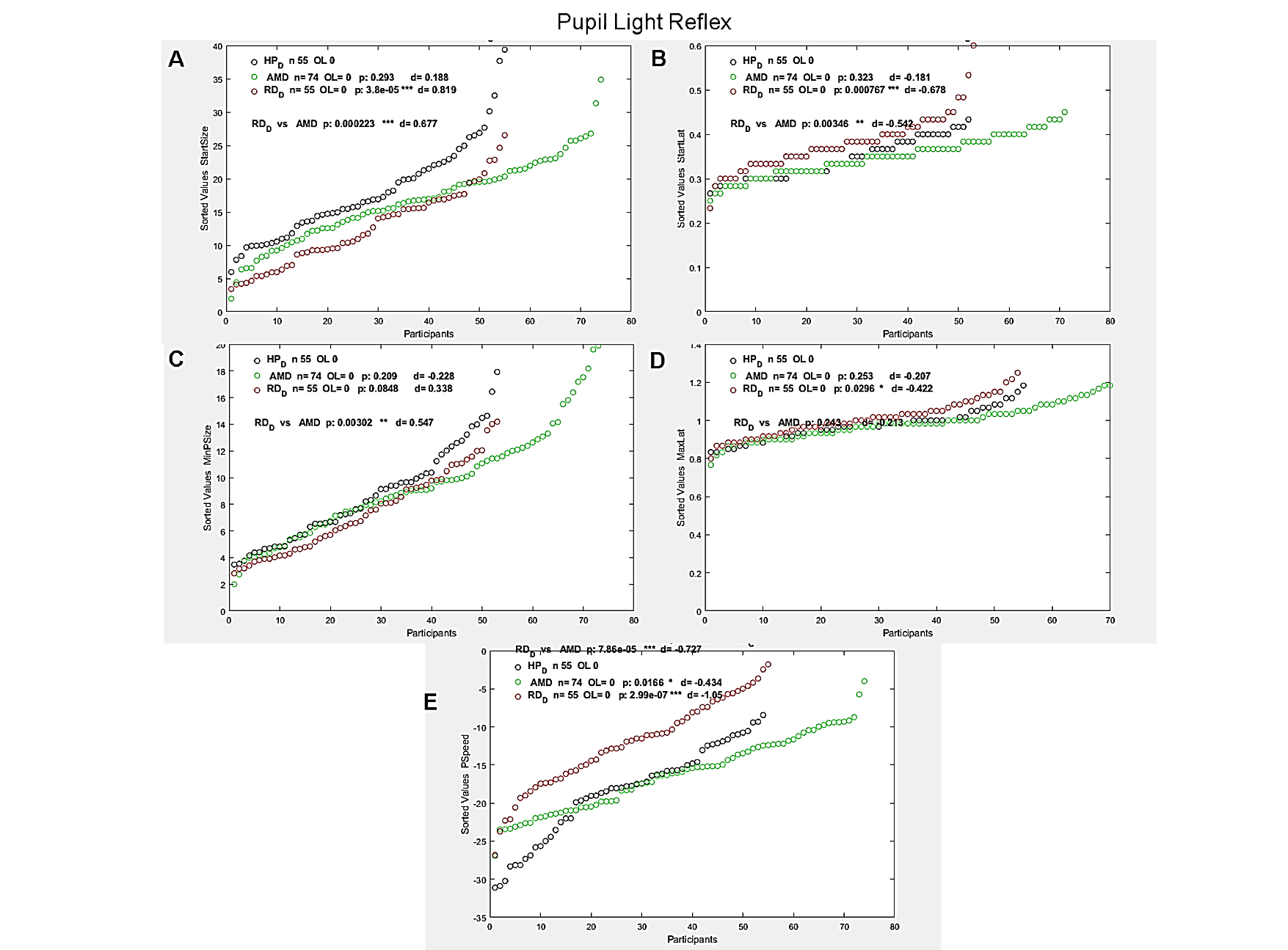


Supplementary Figure 2: Sorted distributions of the variables characterizing the Pupil Light Reflex, after correction for age using the HP data. A. Base line pupil size (*StartSize*, A.U.). B. Latency of starting constriction (*StartLat*); C. Latency to reach minimum size (*MaxLat*). D. Minimum pupil size at maximum constriction (*MinPSize*, A.U.). E. Average constriction speed (*Pspeed*). HP, black symbols; AMD, green symbols, DR, red symbols. MLA (N=18) blue symbols are also shown


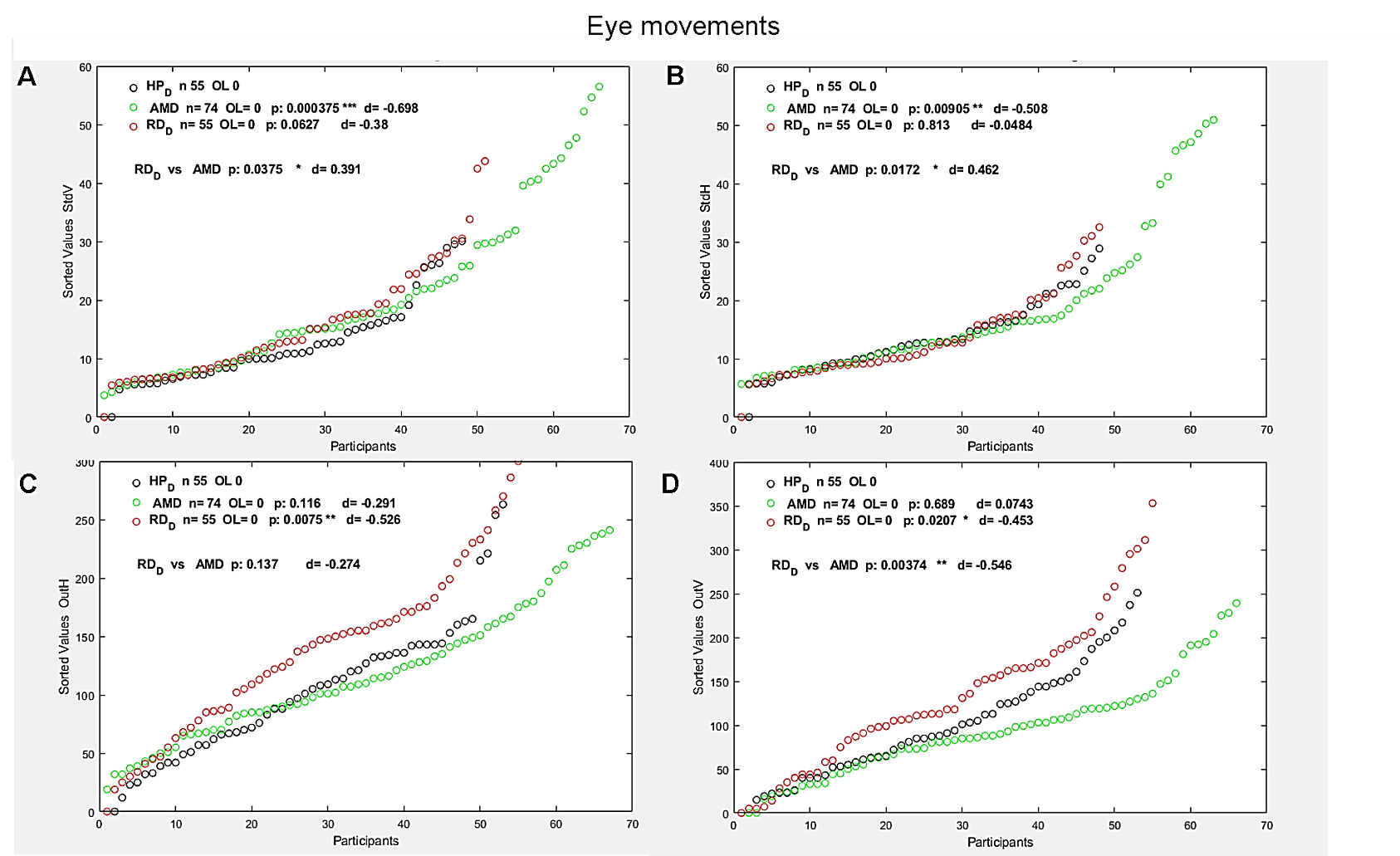


Supplementary Figure 3: Sorted distributions of variables characterizing eye movements. A. Standard deviation of horizontal eye positions during mPFT. B. Standard deviation of vertical eye positions during mPFT. C. Horizontal number of outlier eye positions (eye data greater than 2 standard deviations from the mean eye position). D. Vertical number of outlier eye positions.


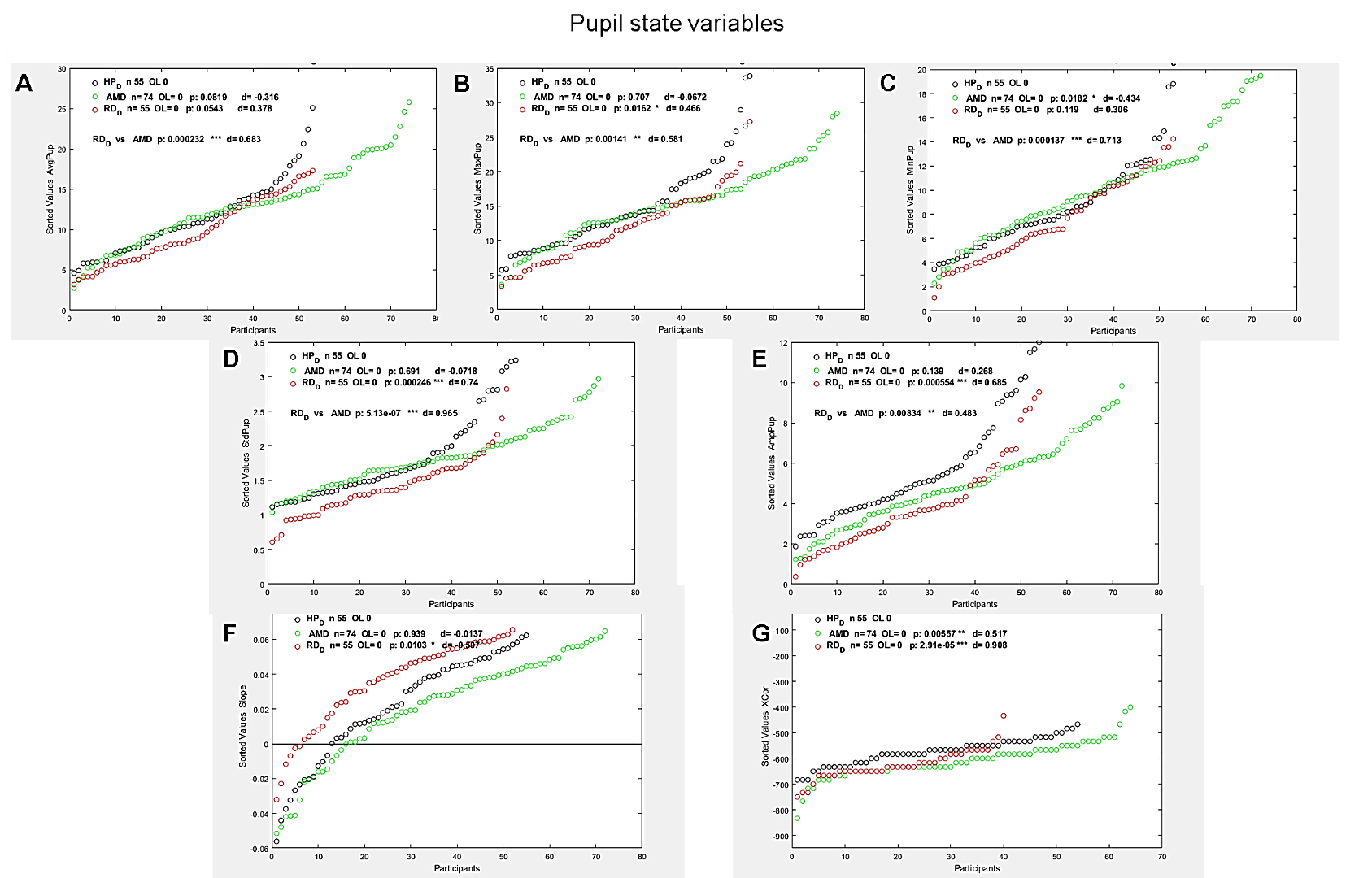


Supplementary Figure 4: Sorted distributions of Pupil State Variables during mPFT. A. Mean pupil size during mPFT (A.U.). B. Mean amplitude of oscillatory responses measured as the difference between maximum and minimum pupil size during mPFT. C. Minimum pupil size during mPFT (A.U.). D.Pupil Standard deviation. E. Standard deviation of pupil responses during mPFT. F. Slope of a linear fit of pupil size over time, assessing pupil drift. G. Cross-correlation between stimulus signal and pupil dynamics (stimulus/response delay in msec.) measured with the Matlab function *xcor*.


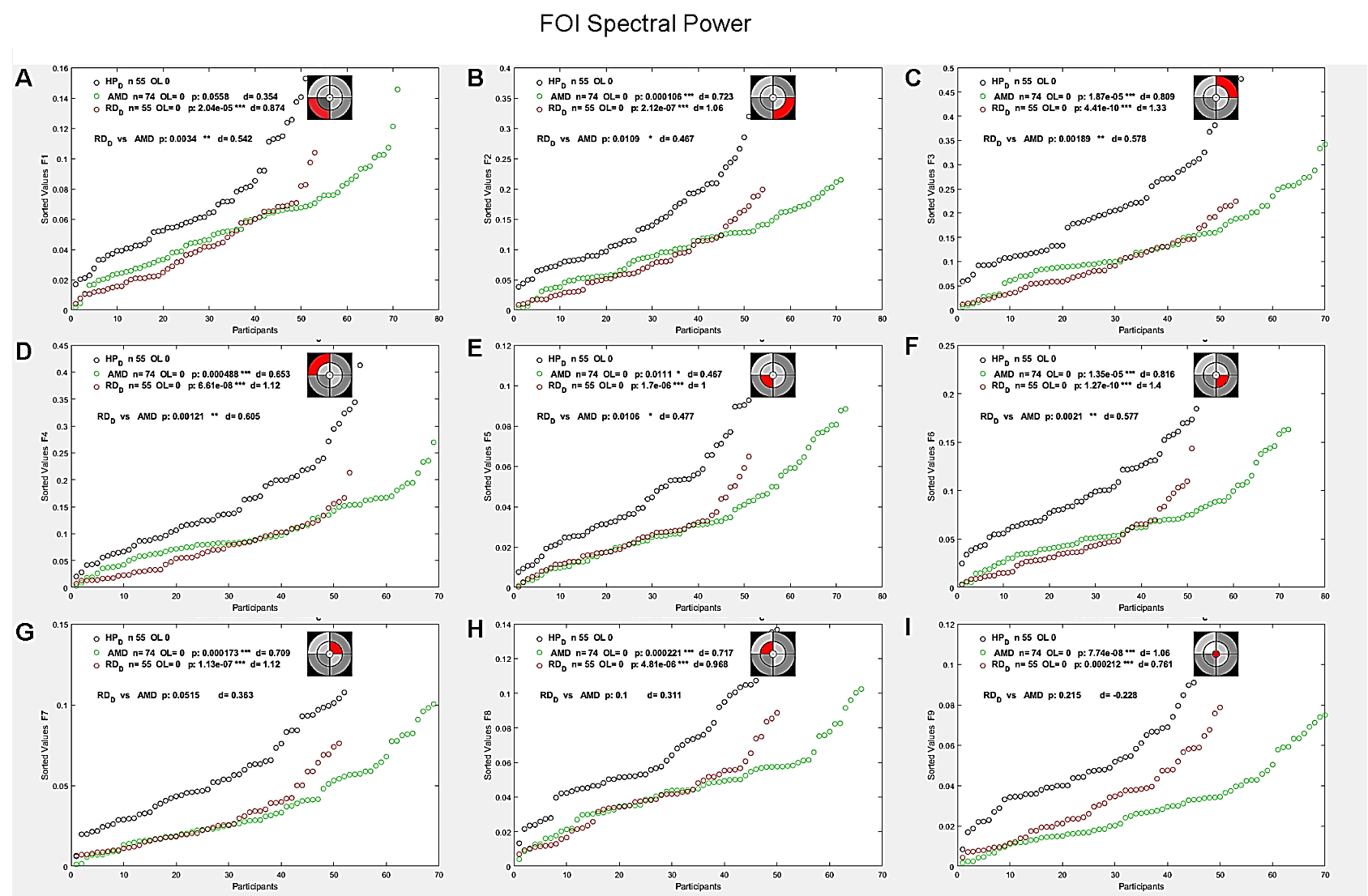


Supplementary Figure 5: Sorted distributions of normalized spectral power for each FOI. Normalization was performed by multiplying each FOI power by the square of the frequency. This was done to compensate for the 1/F power distribution of the FFT spectrum. A-I. Distribution of spectral power for each FOI and group. The insets indicate which sector of the stimulus was analyzed.


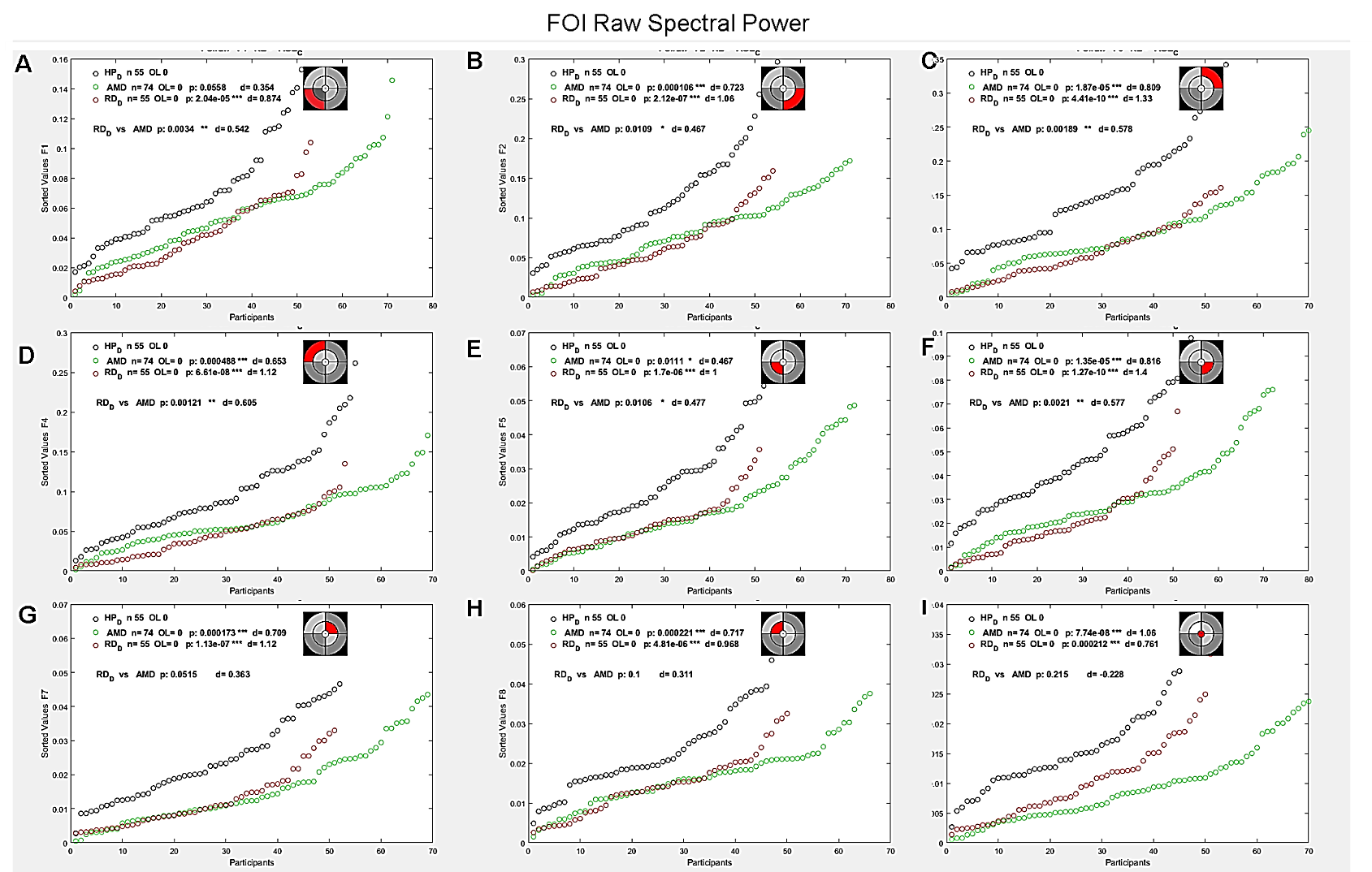


Supplementary Figure 6: Same as Figure 5 for the raw spectral power distributions.


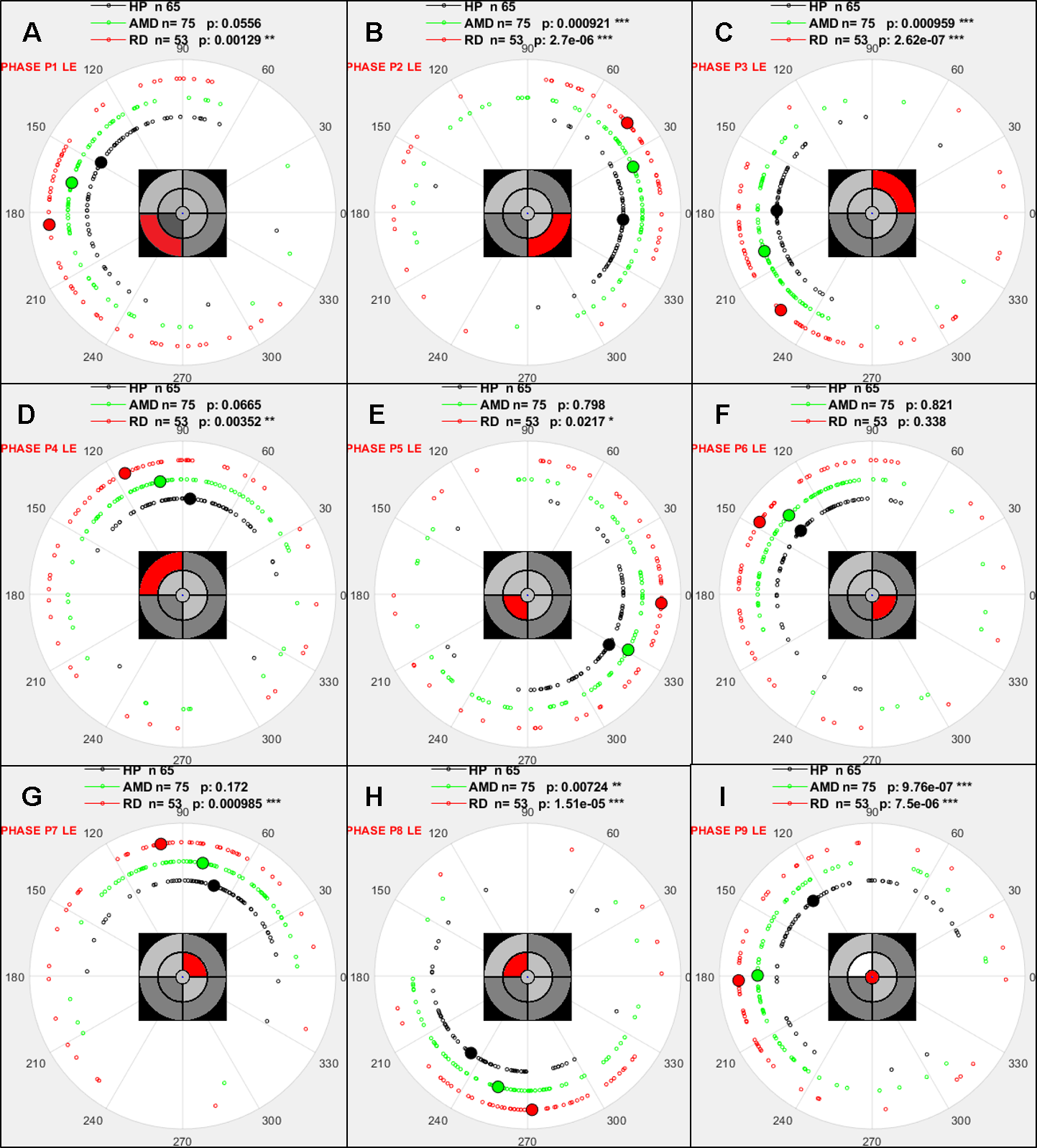


Supplementary Figure 7: Phase distributions for each FOI of the left eye. A-I. Polar plots of phase with different radii for each group to facilitate readability. Statistics were computed using a library of routines for circular statistics (Berens, P., 2009). Filled markers indicate the circular mean. Insets indicate which sector of the stimulus was analyzed.


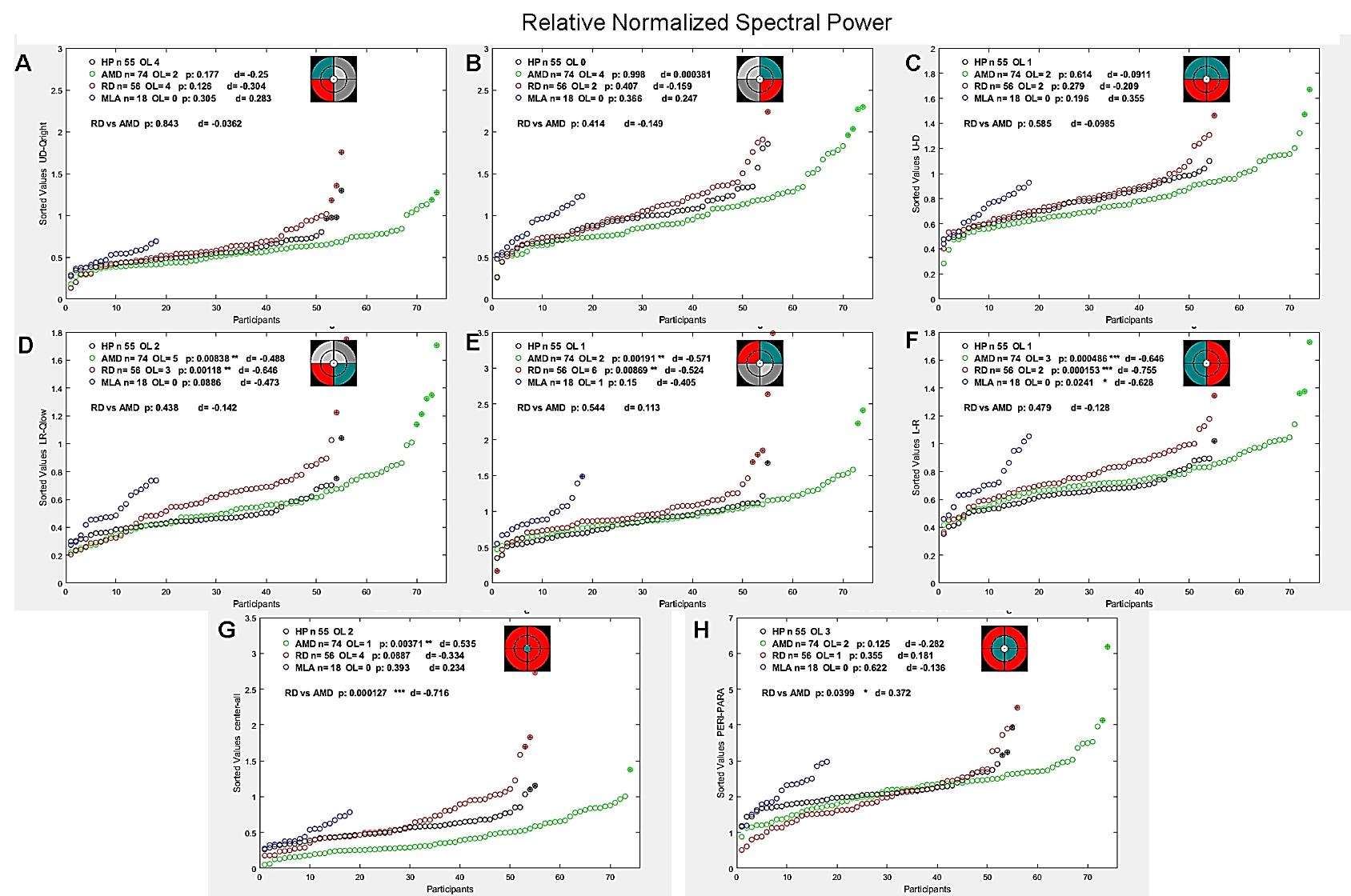


Supplementary Figure 8: A-G Sorted distributions of relative power, computed as the ratio of power between quadrants and hemifields (up/down, left/right), and the ratio of central power to the mean power of all other sectors. Insets show the calculated ratios. Note the significant difference for AMD in Figure G, not seen for DR patients.


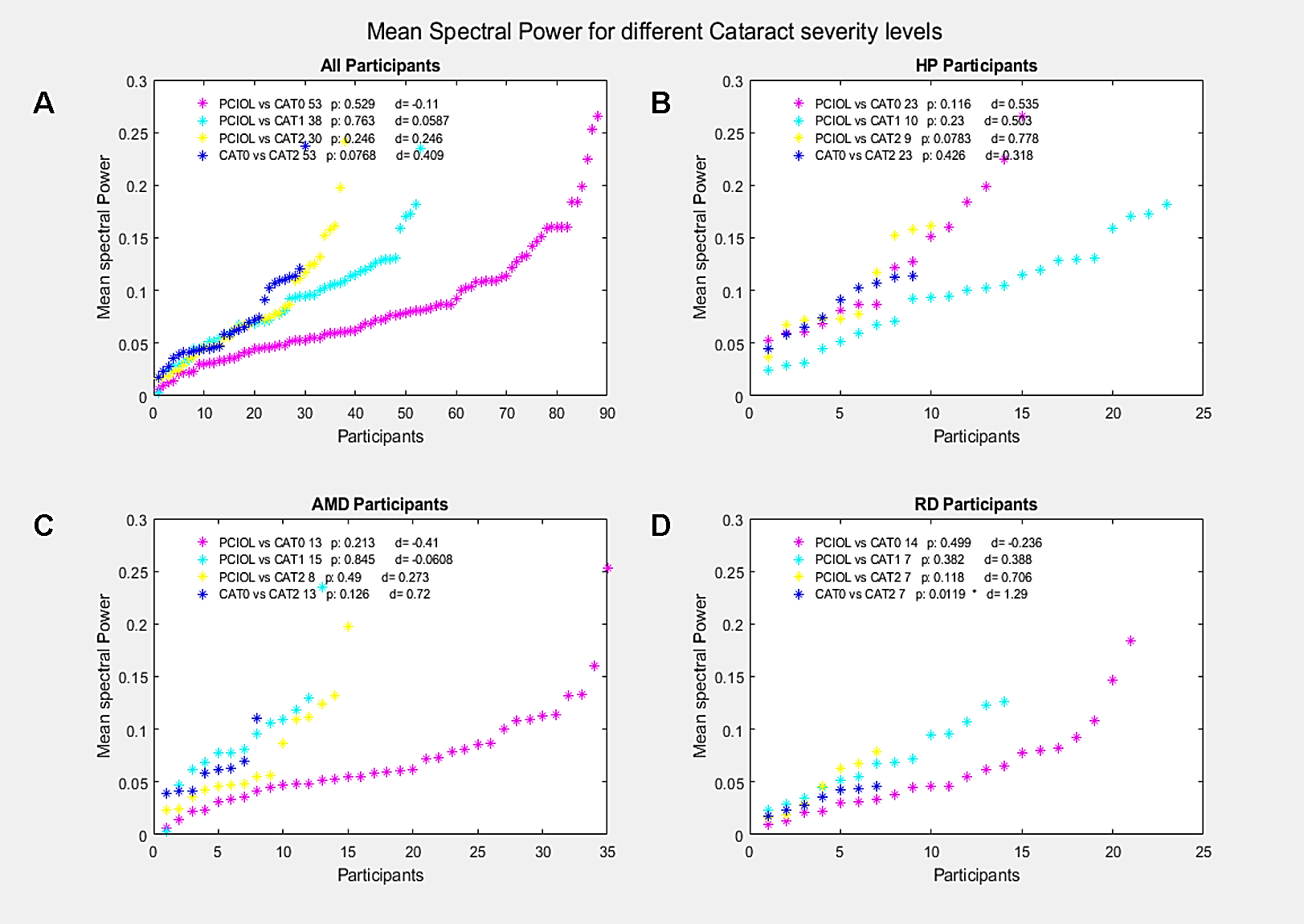


Supplementary Figure 9: Relationships between Cataract Severity and Spectral Power. A. Sorted distribution of spectral power for each participant and different cataract severities. CAT0, no cataract (magenta), CAT1, moderate (cyan), CAT3, severe. PCIOL are individuals who underwent lens implantation surgery. B. Same distributions for HP only. C. Same distributions for the AMD group. D. Same distributions for RD group. Insets show results of Student's t-test comparing mean spectral power between the different severity levels. None of these tests were significant (p>0.05), except for RD patients and the comparison of CAT0 and CAT2 (p<0.05; d=1.29).


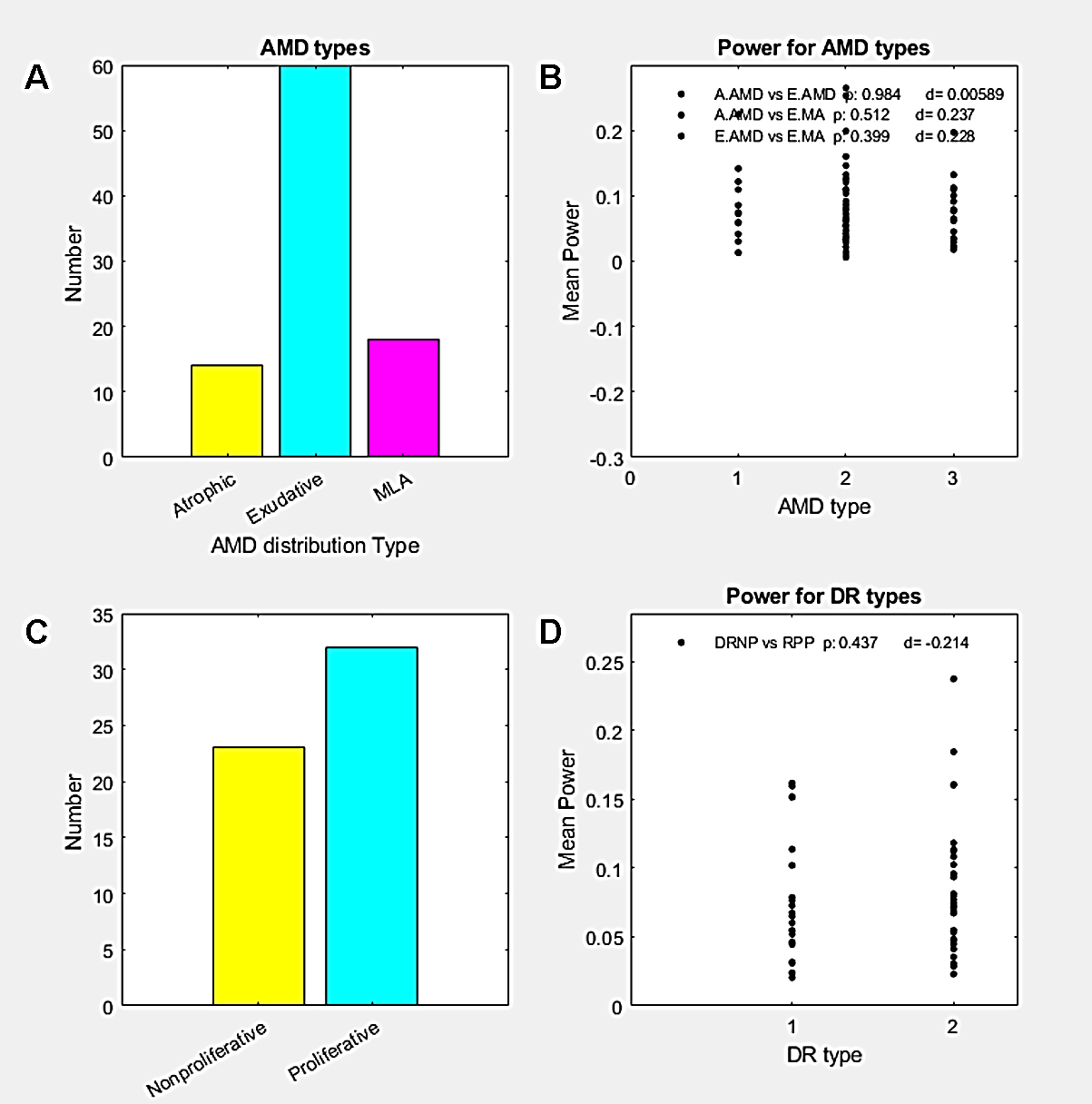


Supplementary Figure 10: Mean spectral power as a function of AMD and DR types. A. Distribution of AMD types in the AMD group. B. Mean spectral power as a function of AMD type. No significant differences were found (all p>0.05). C. Distribution of DR types in the DR group. B. Mean spectral power as a function of DR type. No significant difference was found (p>0.05).


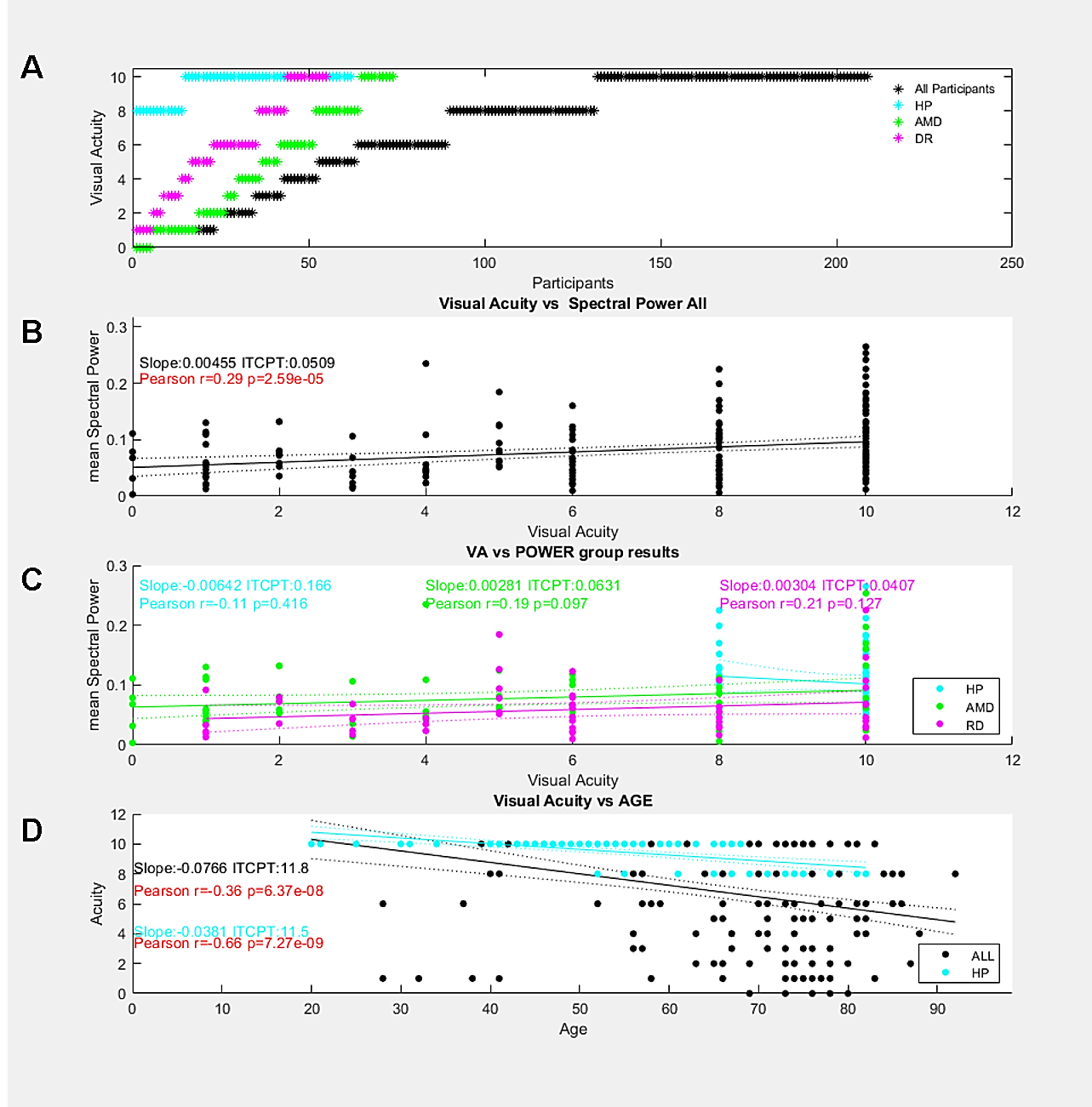


Supplementary Figure 11: Relationships between visual acuity and mean spectral power. A. Visual acuity distributions for all subjects (black symbols) and for each group (HP, cyan; AMD, green; DR, magenta). B. Mean spectral power as a function of visual acuity of all participants showing a significant correlation (Pearson's correlation coefficient, r=0.29; p<0.001). C. Mean spectral power as a function of visual acuity for each group. Pearson's correlation coefficient shows no correlation within each group (all p>0.05). D. Visual acuity as a function of age for all participants (black) and the HP group (cyan). Significant correlations between age and visual acuity are found (all p<0.01).


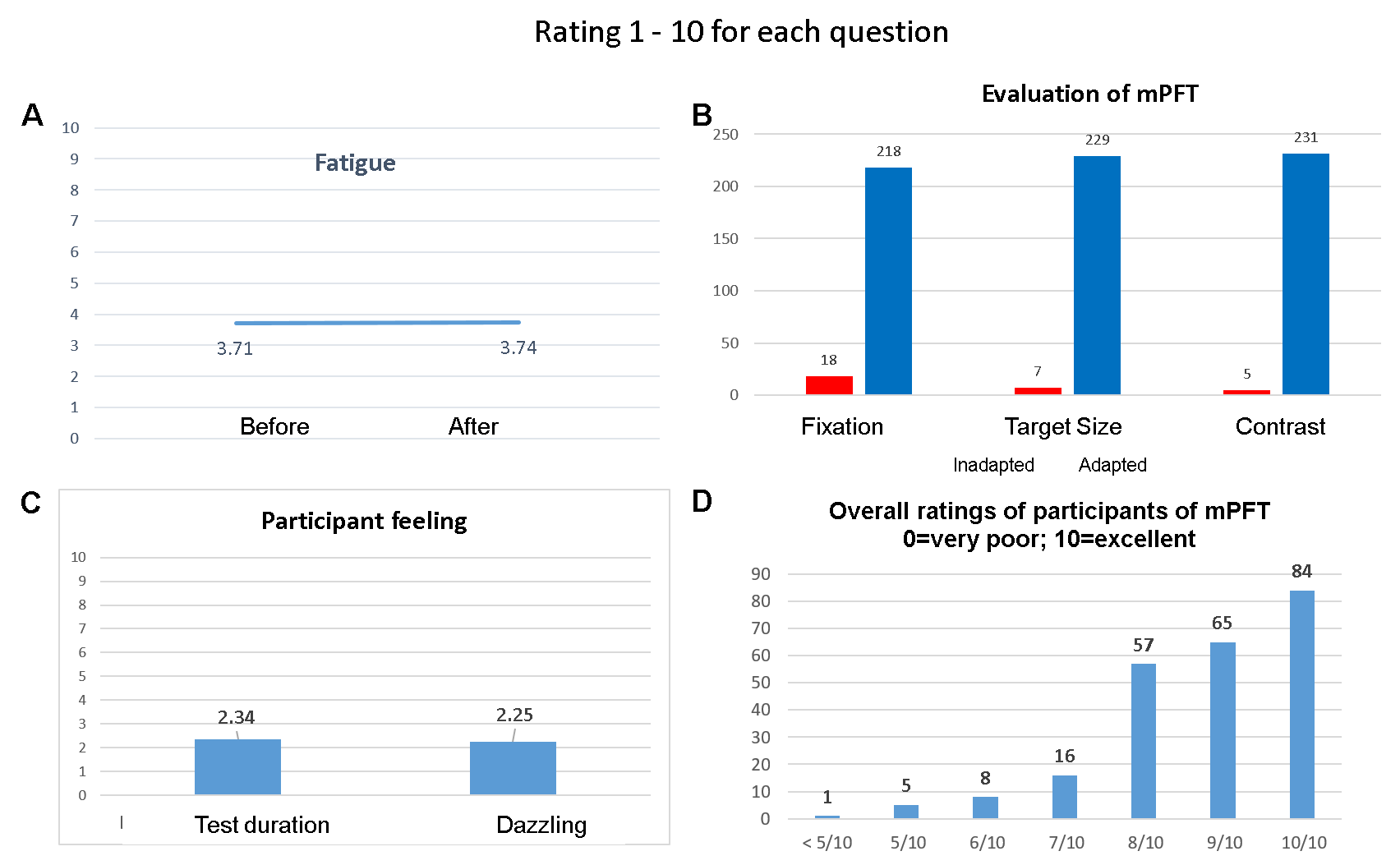


Supplementary Figure 12: Answers to the questionnaire evaluating acceptability and features of mPFT. A. Participants were asked to rate their fatigues before and after the mPFT test B. Evaluation of mPFT characteristics: fixation difficulty; size of fixation point; Glare related to stimulus contrast. C. Evaluatio of test duration and of glare; Subjective rating of the mPFT test. Overall, the mPFT test is well accepted and not judged difficult.

Supplementary Movie # 1: Excerpt of the mPFT stimulus showing luminance modulations of each of 9 sectors with incommensurate temporal frequencies.
